# Clinical Phenotypes, Neuroimaging, and Outcomes in Prolonged and Persistent Non-Motor Migraine Aura: A Systematic Review and Episode-Level Synthesis

**DOI:** 10.3390/neurolint18100197

**Published:** 2026-10-21

**Authors:** Assel Meiramova, Bayan Ainabekova, Yernar Tursynbet, Zhanar Akhmetova

**Affiliations:** 1 Scientific Department, Art Science Innovation Center LLP, 010005 Astana, Kazakhstan; meiramova.as@amu.kz; 2 Department of Internal Medicine No. 2, Astana Medical University, 010000 Astana, Kazakhstan; ainabekova.b@amu.kz; 3 Organizational and Methodological Department, National Center for Sports Medicine and Rehabilitation, 010017 Astana, Kazakhstan; yernartursynbet@gmail.com; 4 Department of Rehabilitation and Sports Medicine, Astana Medical University, 010000 Astana, Kazakhstan

**Keywords:** migraine with aura, prolonged aura, persistent aura without infarction, diffusion-weighted imaging, cerebral perfusion, neuroimaging

## Abstract

Background/Objectives: Prolonged and persistent migraine aura may mimic cerebral ischemia, but its clinical course and neuroimaging features are poorly characterized. We synthesized episode-level evidence on non-motor aura lasting more than 60 min without reported established infarction. Methods: This systematic review (International Prospective Register of Systematic Reviews [PROSPERO] CRD420261461238) followed the Preferred Reporting Items for Systematic Reviews and Meta-Analyses (PRISMA) 2020 guidelines. PubMed, Scopus, and Google Scholar were searched from inception to August 2026 and the searches were rerun with an extended strategy on 5 October 2026, supplemented by backward and forward citation searching. Eligible reports provided individual-episode clinical data and temporally related neuroimaging. Owing to publication selection, heterogeneous reporting, and clustering, the synthesis was descriptive. Results: Forty-eight studies described 80 episodes in 79 individuals (49 prolonged; 31 persistent). Visual aura occurred in 63/80 episodes. Although magnetic resonance imaging was performed in 77/80 episodes, patient-level informative diffusion imaging was available in only 28/80 and imaging-based perfusion data in 28/80. Five of the 28 episodes with informative diffusion imaging were diffusion-positive (17.9%); the acute apparent diffusion coefficient (ADC) was reduced in 3, non-restricted in 1, and unreported in 1, and all diffusion-weighted imaging (DWI) abnormalities resolved on follow-up. Unusual imaging findings are more likely to be published; this proportion is therefore hypothesis-generating rather than a frequency estimate. In the longer-follow-up stratum, aura improvement was reported in 36/36 prolonged and 18/22 persistent episodes, with complete resolution in 35/35 and 15/22, respectively; a nerve-block series with a fixed 24-h assessment (14 episodes) was analyzed separately. Conclusions: Published cases show heterogeneous phenotypes and cannot support estimates of prognosis or treatment effectiveness. Standardized prospective data collection should incorporate symptom-level duration, imaging timing, DWI/ADC reporting, treatment timing, and protocol-defined follow-up.

## 1. Introduction

Migraine with aura is characterized by fully reversible focal neurological symptoms that usually develop gradually and precede or accompany headache. In the International Classification of Headache Disorders, third edition (ICHD-3), typical aura comprises visual, sensory, and/or speech–language symptoms; migraine with brainstem aura is classified separately. Each individual aura symptom usually lasts 5–60 min [1]. Visual phenomena are the most common, followed by sensory and speech–language symptoms [2]. This temporal and phenomenological pattern is clinically important because acute focal deficits also require exclusion of cerebrovascular disease.

A substantial minority of aura symptoms exceed 60 min. In prospective diary studies, 17.6% of recorded auras lasted longer than one hour [3], and the corresponding symptom-level proportion derived from the data of Viana et al. is approximately 15% [2]. ICHD-3 provides a dedicated code for persistent aura without infarction (1.4.2) when symptoms persist for at least 1 week in a patient with migraine with aura and are typical of previous auras, but no dedicated category exists for episodes lasting more than 60 min and less than 7 days [1]. The earlier syndrome of migraine with prolonged aura was abandoned in the second edition; residual cases are now coded as probable migraine with aura with the atypical feature specified [1]. We therefore use prolonged (>60 min but <7 days) and persistent (≥7 days) as operational duration descriptors rather than assuming that every episode fulfills all criteria for ICHD-3 1.4.2 [4].

The principal diagnostic concern is differentiation from ischemic stroke or transient ischemic attack (TIA). Migraine aura may be accompanied by reversible perfusion abnormalities and, rarely, transient diffusion-weighted imaging (DWI) signal changes with either reduced or elevated apparent diffusion coefficient (ADC) values [5]. Interpretation therefore depends on the clinical sequence, vascular and structural imaging, ADC maps, acquisition timing, and follow-up. Reversible cerebral vasoconstriction syndrome (RCVS), arterial dissection, seizure, hemiplegic migraine, and other secondary causes must also be considered [6,7,8].

Evidence for prolonged and persistent non-motor aura consists predominantly of case reports and small case series. Earlier narrative reviews used heterogeneous definitions and did not report registered review methods, reproducible selection processes, or episode-level assessment of reporting completeness [4]. Consequently, the frequency and meaning of diffusion and perfusion abnormalities, the range of treatments used, and the reported clinical course remain uncertain. A systematic synthesis is needed to distinguish descriptive signals from conclusions that this publication-selected literature cannot support.

We therefore systematically reviewed published episodes of prolonged and persistent non-motor aura without established infarction. The objectives were to characterize clinical phenotypes and duration; summarize structural, diffusion, and perfusion imaging; describe treatment exposures without inferring efficacy; and evaluate the completeness and interpretability of reported outcomes.

## 2. Methods

### 2.1. Protocol and Registration

This review was registered with PROSPERO, the International Prospective Register of Systematic Reviews (CRD420261461238; version 1.0 published on 26 July 2026) and reported in accordance with the Preferred Reporting Items for Systematic Reviews and Meta-Analyses (PRISMA) 2020 guidelines [9]. Following completion of the review, the registration record was updated to document methodological clarifications and departures (version 2.0 published on 28 August 2026; version 2.1 published on 1 September 2026). No separate full protocol was prepared.

### 2.2. Eligibility Criteria

Eligibility was framed using the Condition, Context, and Population (CoCoPop) structure rather than the Population, Intervention, Comparator, and Outcome (PICO) framework because the review addresses the presentation, imaging correlates, and course of a condition rather than the effect of an intervention; no comparator was specified. Eligibility was assessed at the level of the individual aura episode, not at the level of the patient or the publication. The full inclusion and exclusion criteria are presented in Table 1. Episodes were operationally classified as prolonged (>60 min but <7 days) or persistent (≥7 days). These are duration descriptors and not ICHD-3 diagnostic categories: episodes classified as persistent were not required to fulfill every criterion for ICHD-3 1.4.2 persistent aura without infarction, and neither descriptor implies migrainous infarction, which was itself an exclusion. Duration was classified based on a single continuous non-motor symptom rather than the diagnostic label used by the authors of the primary report. A duration above 60 min had to be stated explicitly or follow arithmetically from the reported course; a stated duration of exactly one hour did not qualify. Temporal linkage between neuroimaging and the index episode was adjudicated for eligibility at the report level: it was accepted when imaging was explicitly obtained during the index episode, or when the source placed it within the acute assessment of that episode or while symptoms persisted (contextual temporal attribution). Episodes were excluded when imaging was explicitly obtained outside the episode or as an interictal work-up, or when its timing could not be resolved from the complete source. Operational full-text rules introduced during the revision (exclusion of acute confusional migraine; exclusion of episodes that the source authors themselves attributed to a non-migraine cause, whereas an alternative cause that could merely not be ruled out led to retention with a flag; and exclusion of reports predating clinical computed tomography) are recorded with their dates in Supplementary Table S7.

### 2.3. Information Sources and Search Strategy

PubMed and Scopus were searched from inception to 23 August 2026 and Google Scholar to 24 August 2026, without date or language filters. The reference lists of the included studies were checked for additional eligible reports. In response to peer review, the searches were rerun on 5 October 2026 with an extended strategy: PubMed with Medical Subject Headings and additional synonym blocks (including aura status, status migrainosus, stroke mimic, aphasia, and terms for reversible imaging findings), Scopus with an explicit index-term component (the original Scopus strategy searched title, abstract, and keyword fields and was extended with an explicit INDEXTERMS component), and Google Scholar with thirteen archived allintitle queries. Forward citation tracking (Scopus cited-by searches of all included reports and Google Scholar cited-by searches of reports not indexed in Scopus) and backward citation tracking of the newly included reports were added; citation tracking was completed on 8 October 2026. Complete search strings, dates, fields, yields, and Google Scholar screening depths are provided in Supplementary Table S1 in accordance with the PRISMA extension for literature searches (PRISMA-S) [10].

### 2.4. Selection Process

All identified records were collated, and duplicates were removed via DOI and title matching. The Google Scholar branch was screened manually, record by record, with a written log kept for each run. Title and abstract screening of the original searches was performed by a single reviewer (Z.A.), as registered. In the database branch, records were first sorted into include, uncertain, or exclude categories by applying a rule-based (non-artificial-intelligence) keyword triage to the title text; of the 756 unique database-branch records, 312 were excluded by this triage and 444 were read individually and proceeded to retrieval. The figure of 759 given in the originally submitted version of this section referred to the all-branch July 2026 screening log rather than to the database branch of the flow diagram; the two denominators are now reported separately. In response to peer review, all keyword-excluded records were subsequently re-screened individually and independently by two reviewers (Z.A. and A.M.), blinded to each other’s decisions, against the registered criteria; where no abstract existed, a record could be excluded on its title alone only if the title unambiguously met an exclusion criterion, and a record proceeded to full-text assessment if either reviewer selected it. One report identified through this re-screen (Parks et al.) proved eligible and was recovered; two further reports set aside in error had been recovered earlier. In the original review, full-text reports were independently assessed by two reviewers (Z.A. and A.M.), disagreements were resolved through discussion and consensus, and no automation tool was used for full-text eligibility decisions, data extraction, or critical appraisal. In the October 2026 update, title and abstract screening of the 2957 newly identified records and the full-text assessment of the 313 retrieved reports were performed by one reviewer (Z.A.) with the aid of a generative artificial intelligence tool (Claude Opus 5.5, Anthropic; accessed via claude.ai, 2026). The tool was used to retrieve and extract verbatim passages from the full texts, to draft a provisional assessment of each record against the eligibility criteria, to flag borderline records, and to maintain the decision and change logs; it did not make eligibility decisions, and the final decision for every record was made by the reviewer, who consulted the full text whenever the evidence was insufficient, ambiguous, or the record was borderline. To verify this process, a blinded stratified sample of 49 full-text records (all candidate inclusions, all borderline records, and a random sample of exclusions) was assessed independently by the second reviewer (A.M.) without artificial intelligence tools and without access to the first reviewer’s decisions. Agreement in the independent phase was 87.8% (43/49; Wilson 95% confidence interval (CI) 75.8–94.3%; Cohen’s kappa 0.62; prevalence-adjusted bias-adjusted kappa (PABAK) 0.76; Gwet’s first-order agreement coefficient (AC1) 0.82), and all discordant records were resolved by discussion of the source full texts, with each consensus decision and its rationale recorded. This single-reviewer, verification-based procedure for the update round is a documented departure from the registered dual full-text assessment (Supplementary Table S4). Each excluded full-text report was assigned one primary reason from the registered set of exclusion reasons; the ordering of reasons was finalized for consistent reporting and did not alter any eligibility decision. The excluded reports, decision hierarchy, and cross-branch reconciliation are provided in Supplementary Table S3; three reports could not be retrieved.

### 2.5. Data Collection and Data Items

One reviewer extracted data using a piloted standardized form, and a second reviewer checked every field against the source report (Z.A. and A.M.). Study investigators were not contacted for additional data. Included reports published in languages other than English (listed in Supplementary Table S4) were assessed and extracted with the aid of online machine translation. The review had no single primary outcome because it addressed the description of a condition rather than the effect of an intervention. Three outcome domains were sought: aura phenotype and duration; neuroimaging findings, including diffusion status, apparent diffusion coefficient, perfusion, and repeat imaging; and clinical course, defined as aura improvement and complete resolution at the last assessment reported. Improvement and complete resolution were taken as classified by the authors of the primary report and were not re-adjudicated. The extracted variables included study characteristics; age and sex; symptom type and duration; lifetime history of motor aura; headache during the episode; neuroimaging modality, diffusion and ADC findings, perfusion findings, repeat imaging, and radiologic normalization; treatment directed at the aura or attack and the agents given; and outcome at the last reported assessment together with follow-up duration. Separately, and more strictly, we recorded for each included episode whether at least one neuroimaging study was documented as having been performed while qualifying aura symptoms were present (yes, no, or unclear); this descriptive variable used only explicit source-level temporal information, presumed timing was coded as unclear, and it was not used to determine eligibility.

### 2.6. Critical Appraisal Methods

Methodological reporting was appraised with the design-specific Joanna Briggs Institute (JBI) checklists for case reports, case series, and analytical cross-sectional studies [11,12]. One reviewer performed the appraisal, and a second reviewer checked each judgment (Z.A. and A.M.). Each item was mapped to extraction fields and rated Yes only when the required information was documented; non-adjudicable items were rated Unclear. In the statistical analysis, items for purely descriptive reports were marked as not applicable. Because JBI does not recommend converting checklist responses into summary scores or universal low/moderate/high categories, appraisal is reported item by item in Supplementary Table S2 and interpreted according to recurring domain-level limitations.

### 2.7. Synthesis Methods

A structured descriptive synthesis was performed at the episode level. Because episodes were clustered within studies of unequal size and, in one instance, within a patient, observations were not treated as independent. Categorical variables are reported as counts and proportions with explicit available-case denominators, whereas continuous variables are summarized using medians and interquartile ranges (IQRs) or ranges where sufficiently reported. Missing values were not imputed. Descriptive statistics and two figures were produced in Python 3 using numpy, matplotlib, and openpyxl; the analysis code and its dependency specification are provided in Supplementary File S1, which regenerates the reported counts, all main-text tables, Supplementary Table S8, and both figures from the frozen dataset in Supplementary Table S6. Prolonged and persistent groups are displayed side by side for descriptive purposes only. Formal between-group tests and meta-analyses were not performed because the reports lacked comparators, and definitions, imaging timing, and outcome ascertainment were heterogeneous. Sensitivity analyses are reported in Supplementary Table S8 and comprise the two prespecified scenarios (exclusion of the largest series and exclusion of the four transient DWI-positive episodes admitted under the registration amendment) together with ten further scenarios specified during revision, including exclusion of the 26 episodes flagged for weaker supporting evidence and leave-one-study-out analyses. Outcomes were analyzed in two prespecified strata: episodes with longer or variable follow-up, and the nerve-block series with a fixed 24-h assessment, which is reported separately. Formal assessment of selective non-reporting was not undertaken because protocols and prespecified outcomes were unavailable for most primary reports. Certainty was not graded because the uncontrolled case-based evidence provided no estimable comparative effect.

### 2.8. Amendments to the Registered Record

Differences between the initial PROSPERO record and the review as conducted are documented in Supplementary Table S4. Three departures were restrictive: conference-only reports, one unpublished non-peer-reviewed report, and episodes with documented ocular motor palsy were excluded [13]. Other amendments broadened eligibility or clarified methods: Scopus was added before screening, the registered English/Russian restriction was removed, and transient DWI abnormalities were permitted when established infarction was excluded and reversibility was documented. The allocation of JBI appraisal instruments was specified after registration and applied consistently by report design [14,15]. The restrictive departures remain a potential source of selection bias. Further departures documented during revision (Supplementary Table S4) comprise the extended update search of 5 October 2026 with forward citation tracking; the single-reviewer, artificial-intelligence-assisted assessment of the update records verified by a blinded second-reviewer sample (Section 2.4); operational full-text rules recorded with their dates (Section 2.2; Supplementary Table S7); re-adjudication of every previously included episode against the registered criteria, with each decision reported in Supplementary Table S7; recoding of the largest series to its constituent patients; and separate analysis of the fixed 24-h nerve-block stratum.

## 3. Results

### 3.1. Study Selection and Characteristics

**Figure 1:**
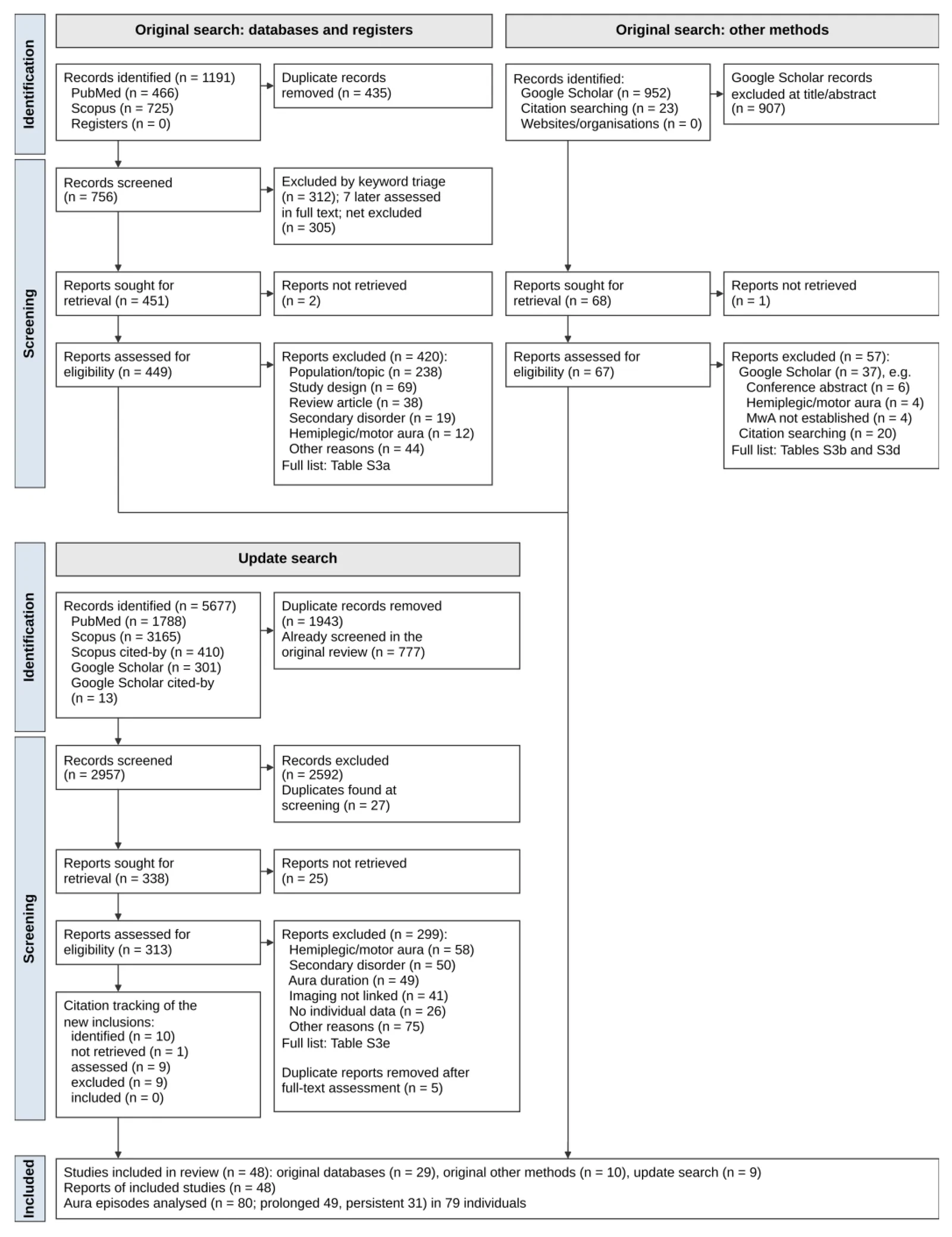
Preferred Reporting Items for Systematic Reviews and Meta-Analyses (PRISMA) 2020 flow diagram [9] of the original and update searches. Full exclusion reasons are listed in Supplementary Table S3. MwA, migraine with aura.

Database searching identified 1191 records, and other methods identified 975 branch-unique records; the update search of 5 October 2026 identified 5677 additional records, of which 3734 were unique and 2957 had not been screened previously. After duplicate removal, screening, full-text assessment, and re-adjudication of every previously included episode against the registered criteria, 48 studies contributing 80 eligible episodes in 79 individuals were included: 29 from the original database branch, 10 from other methods, and 9 from the update search. Twenty reports included in the originally submitted version were excluded on re-adjudication against the registered criteria [16,17,18,19,20,21,22,23,24,25,26,27,28,29,30,31,32,33,34,35]; each decision, with its operational rule and date, is documented in Supplementary Table S7. Figure 1 shows the full study flow, and Supplementary Table S3 lists the full-text exclusions and reconciles four records screened in an August Google Scholar run but assigned to another identification branch. In the dual re-screen of the keyword-excluded records, the reviewers agreed on 299 of 304 (98.4%; Wilson 95% CI 96.2–99.3%); Cohen’s kappa was uninformative (−0.005) because full-text selection was rare (prevalence paradox), PABAK was 0.967, and Gwet’s AC1 was 0.983. Five records proceeded to full-text assessment: one (Parks et al.) was included, and none of the remaining four met the eligibility criteria. In the blinded verification of the update-round full-text assessment, agreement in the independent phase was 87.8% (43/49), and all discordant records were resolved by consensus (Section 2.4).

The 48 studies were published from 1991 to 2026 and represented 18 countries. At the study level, 31 were case reports, 15 were case series, and 2 were cohort or partial individual-patient-data studies; at the episode level, 39 observations came from case series, 32 from case reports, and 9 from cohort or partial individual-patient-data studies. Table 2 summarizes the study characteristics, and Supplementary Table S5 details the investigations and treatment.

Study contributions were unequal: 38 reports contributed 1 episode, whereas the largest prospective series contributed 14/80 (17.5%). Reporting completeness varied by variable, and available-case denominators are therefore shown for every estimate in Table 3, Table 4 and Table 5.

### 3.2. Clinical Characteristics of the Aura Episodes

**Figure 2:**
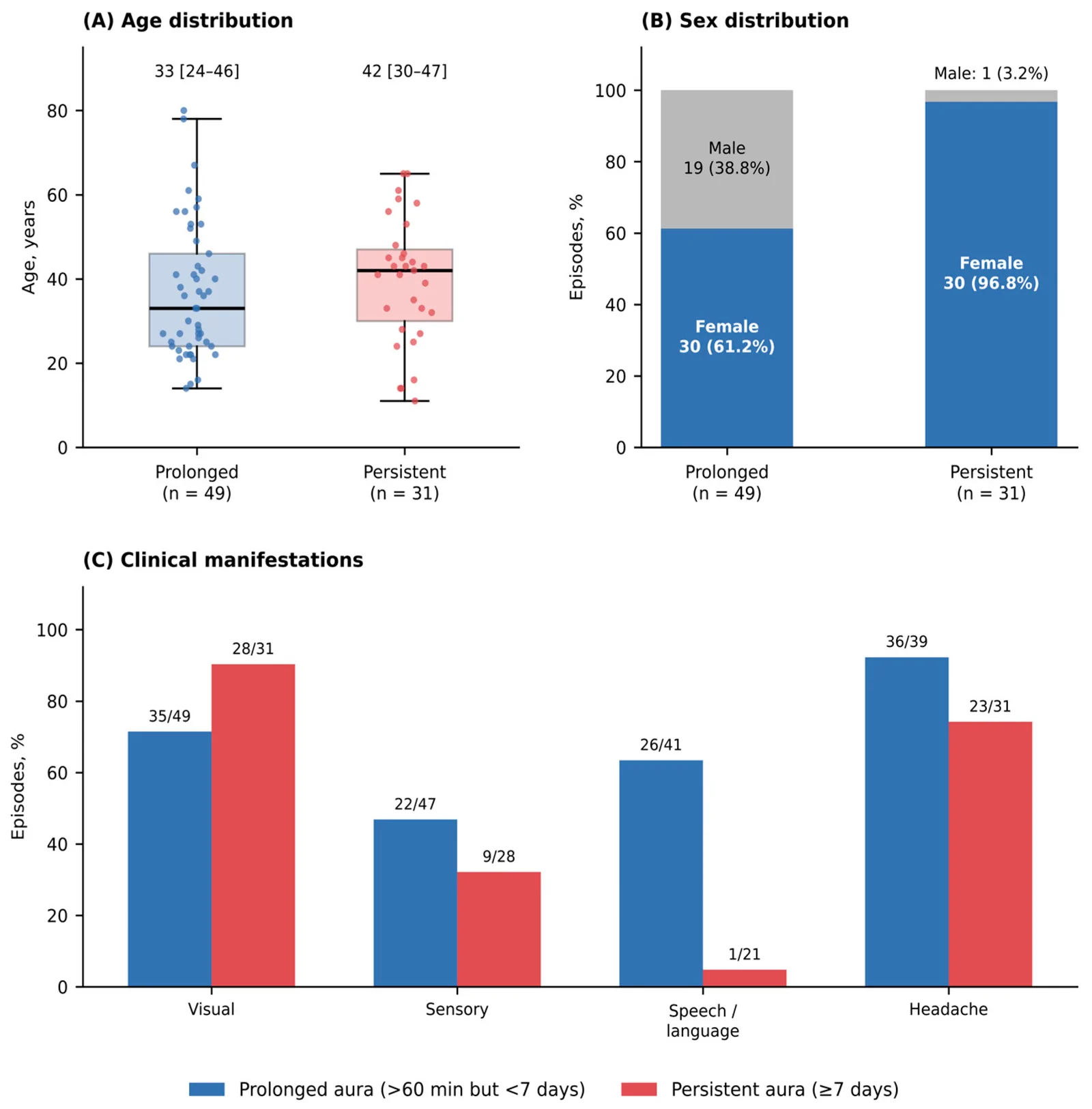
Clinical characteristics of prolonged and persistent non-motor migraine aura. (**A**) Age distribution. (**B**) Sex distribution by episode. (**C**) Visual, sensory, and speech–language aura, and headache presence. Definite brainstem aura is reported in Table 3 (0/57 assessable episodes); no episode fulfilled the strict ICHD-3 1.2.2 requirement. Percentages use available-case denominators; age is median with interquartile range. Categories are shown descriptively, without hypothesis testing. Panels (**A**–**C**) correspond to the variables reported in Table 3.

Among 80 episodes (49 prolonged; 31 persistent), visual aura was reported in 63 (78.8%), sensory aura in 31/75 available episodes (41.3%), and speech–language aura in 27/62 (43.5%). No episode fulfilled the strict ICHD-3 1.2.2 requirement for definite brainstem aura (0/57 assessable episodes). The median age was 37 years (IQR 25–46.5; n = 80) overall, 33 years (IQR 24–46) in prolonged episodes, and 42 years (IQR 30–47) in persistent episodes; additionally, 60/80 episodes occurred in females. Headache accompanied 59/70 episodes with ascertainable headache status (84.3%). An established or retrospectively adjudicable diagnosis of migraine with aura was an eligibility requirement for every episode; in 26 of 80 episodes the supporting evidence was weaker (for example, prior attacks known from history alone), and these episodes were flagged for a prespecified sensitivity analysis (Supplementary Table S8). Not every operationally persistent episode could be confirmed as ICHD-3 persistent aura without infarction. Clinical characteristics by duration group are shown in Table 3 and Figure 2.

**Table 3:** Clinical characteristics of the 80 aura episodes by duration category.

Characteristic	Overall (n = 80)	Prolonged (n = 49)	Persistent (n = 31)
Age, years, median (IQR; min–max)	37 (25–46.5; 11–80)	33 (24–46; 14–80)	42 (30–47; 11–65)
Female, n (%)	60 (75.0)	30 (61.2)	30 (96.8)
Type of aura symptom (ICHD-3 categories), n/N (%)			
Visual	63/80 (78.8)	35/49 (71.4)	28/31 (90.3)
Sensory	31/75 (41.3)	22/47 (46.8)	9/28 (32.1)
Speech or language	27/62 (43.5)	26/41 (63.4)	1/21 (4.8)
Brainstem	0/57 (0.0)	0/36 (0.0)	0/21 (0.0)
Headache during the episode, n/N (%)	59/70 (84.3)	36/39 (92.3)	23/31 (74.2)

IQR, interquartile range. Values are n (%) where the denominator is the whole group and n/N (%) where it is the number of episodes for which the variable was ascertainable; denominators therefore differ between rows, and missing values were not imputed. Age is median (IQR; min–max). The four symptom categories are those listed in criterion B of ICHD-3 1.2 for non-motor, non-retinal aura; they are not mutually exclusive, so the counts do not sum to the group total. Brainstem aura was scored strictly by the ICHD-3 1.2.2 requirement for at least two qualifying symptoms. Aura symptoms outside these categories, such as cognitive disturbance, amnesia, apraxia, and alexia, were described in some reports and are not tabulated.

### 3.3. Neuroimaging Findings

Magnetic resonance imaging (MRI) was reported in 77/80 episodes (96.2%), computed tomography (CT) in 27, single-photon emission computed tomography (SPECT) in 5, positron emission tomography (PET) in 5, and electroencephalography (EEG) in 16. Imaging during ongoing aura symptoms was documented in 53 of 80 episodes (66.2%); it could not be established in 26 (32.5%), including all 14 episodes of the nerve-block series [43], and one episode was imaged only after symptom resolution. All 26 unclear episodes met the eligibility criterion for temporally related imaging through contextual attribution and were retained. Among episodes with a documented structural signal abnormality, symptomatic-phase imaging was documented in 8 of 9, and among episodes with informative diffusion imaging, in 27 of 28. Patient-level informative diffusion imaging was available in 28/80 episodes (35.0%): 23 were DWI-negative, and 5 were DWI-positive. An explicit ADC value accompanying the diffusion result was reported in only a minority of episodes. Of the five positive episodes, one showed a non-restricted (vasogenic) pattern [5]; in the other four, the acute ADC was reduced in three and not reported in one, and these episodes met the eligibility rule because complete radiologic normalization was documented on follow-up [38,39,53,64]. One further included report documented an isolated ADC abnormality with a normal diffusion-weighted image during a prolonged visual aura. Imaging-based perfusion studies were performed in 28/80 episodes (35.0%). As reported by the source authors, the pattern was hypoperfusion only in 15, normoperfusion in 5, both hypo- and hyperperfusion in 3, hyperperfusion only in 2, and not classifiable in 3; heterogeneous methods, reference regions, and acquisition phases nevertheless precluded pooled directional estimates. Repeat imaging was reported in 33 episodes. Among the 21 episodes with an initial neuroimaging abnormality and repeat imaging, complete radiologic normalization was documented in 14 (66.7%), partial improvement without normalization in 3, and a persisting abnormality without documented normalization in 4; the remaining 12 episodes had no initial abnormality. In the 28 episodes with any documented abnormality on episode imaging, the abnormality involved the region expected to generate the dominant aura symptom in 22 of the 24 episodes in which concordance could be assessed (91.7%); one episode was partially concordant, one was discordant, and four were not assessable because the symptoms were non-lateralizing or the side of the abnormality was reported only at cohort level. The single discordant episode was imaged only after aura offset, during the headache phase of the same attack, and its exclusion left no discordant episode among 23 assessable episodes. The temporal relation between imaging and the clinical course was heterogeneous: normalization was documented on imaging obtained after clinical resolution in 12 episodes, the abnormality outlasted clinical resolution in 5, symptoms outlasted radiologic normalization in 2, and the remaining 9 had either no repeat imaging or repeat imaging without documented normalization (Supplementary Table S9). Excluding the four transient DWI-positive episodes admitted under the registration amendment (n = 76) changed the headline outcome proportions by less than one percentage point: aura improvement 50/54 (92.6%) vs. 54/58 (93.1%), and complete resolution 47/54 (87.0%) vs. 50/57 (87.7%) (Supplementary Table S8). The descriptive conclusions of this review were stable across all twelve prespecified and post hoc sensitivity scenarios (Table S8 and Table 4).

**Table 4:** Neuroimaging performed and findings by duration category.

Finding	Overall (n = 80)	Prolonged (n = 49)	Persistent (n = 31)
Imaging method performed, n (%)			
Magnetic resonance imaging	77 (96.2)	46 (93.9)	31 (100.0)
Perfusion imaging (perfusion MRI, CT perfusion, SPECT, or ASL)	28 (35.0)	17 (34.7)	11 (35.5)
Computed tomography, any type	27 (33.8)	23 (46.9)	4 (12.9)
Electroencephalography	16 (20.0)	13 (26.5)	3 (9.7)
SPECT	5 (6.2)	0 (0.0)	5 (16.1)
Positron emission tomography	5 (6.2)	1 (2.0)	4 (12.9)
Imaging performed during qualifying symptoms, n (%)			
Yes	53 (66.2)	32 (65.3)	21 (67.7)
No	1 (1.2)	1 (2.0)	0 (0.0)
Unclear	26 (32.5)	16 (32.7)	10 (32.3)
Transcranial Doppler and/or NIRS, any purpose (not counted as perfusion imaging), n (%)	13 (16.2)	9 (18.4)	4 (12.9)
Diffusion-weighted imaging, n/N (%)			
Patient-level informative DWI available	28/80 (35.0)	19/49 (38.8)	9/31 (29.0)
of these, diffusion-negative	23/28 (82.1)	16/19 (84.2)	7/9 (77.8)
of these, transient DWI hyperintensity with reduced or unreported ADC, radiologic normalization documented	4/28 (14.3)	2/19 (10.5)	2/9 (22.2)
of these, DWI hyperintensity without restricted diffusion (vasogenic pattern), normalized on follow-up	1/28 (3.6)	1/19 (5.3)	0/9 (0.0)
Patient-level informative DWI not available	52/80 (65.0)	30/49 (61.2)	22/31 (71.0)
Repeat imaging and radiologic normalization, n (%)			
Repeat imaging performed	33 (41.2)	25 (51.0)	8 (25.8)
radiologic normalization documented	14 (17.5)	8 (16.3)	6 (19.4)
partial improvement without normalization	3 (3.8)	3 (6.1)	0 (0.0)
abnormality present, normalization not documented	4 (5.0)	3 (6.1)	1 (3.2)
no initial abnormality (normalization not applicable)	12 (15.0)	11 (22.4)	1 (3.2)
Complete radiologic normalization among episodes with an initial abnormality and repeat imaging, n/N (%)	14/21 (66.7)	8/14 (57.1)	6/7 (85.7)
Structural signal abnormality documented (any, irrespective of reversibility)	9 (11.2)	6 (12.2)	3 (9.7)

Imaging method rows are n (%) of the 80 episodes and are not mutually exclusive: one episode may have been examined using several methods. Diffusion rows are n/N (%) using the number of episodes with patient-level informative diffusion imaging, defined as an explicitly reported diffusion result for the individual episode. A proportion computed on all 80 episodes would understate what the informative episodes show because such imaging was unavailable in almost two-thirds of episodes. “Patient-level informative DWI not available” comprises episodes in which diffusion imaging was not performed, was performed but not separately described, or was described too ambiguously to classify. No established infarction was reported in any included episode, by eligibility criterion, and this is therefore not tabulated. Perfusion patterns are reported as stated by the source authors (Section 3.3). ADC, apparent diffusion coefficient; ASL, arterial spin labeling; CT, computed tomography; DWI, diffusion-weighted imaging; MRI, magnetic resonance imaging; SPECT, single-photon emission computed tomography; NIRS, near-infrared spectroscopy; TCD, transcranial Doppler.

### 3.4. Treatment and Outcomes

Treatment directed at the aura or attack was documented in 48 episodes: 72.7% of the 66 episodes with ascertainable treatment status, and 60.0% of all 80 episodes; treatment status was not ascertainable in 14. The exposures recorded most often were greater occipital nerve block (n = 14), valproate (n = 10), lamotrigine (n = 6), corticosteroids and triptans (n = 5 each), and topiramate, furosemide, and non-steroidal anti-inflammatory drugs (NSAIDs) (n = 3 each); these categories were not mutually exclusive. Outcomes were analyzed in two prespecified strata. In the longer/variable follow-up stratum, aura improvement was documented in 54/58 episodes (93.1%) and complete resolution in 50/57 (87.7%): improvement in 36/36 prolonged and 18/22 persistent episodes, and complete resolution in 35/35 and 15/22, respectively. In the nerve-block series with a fixed 24-h assessment (14 episodes), improvement was documented in 11/14 episodes and complete resolution in 5/14; responses followed by recurrence within 24 h were not coded as complete resolution. Follow-up duration was numerically recoverable in 48 of the 66 longer-follow-up episodes (median 14.5 days; IQR 6.75–180; range 3–730; median 14 days in prolonged and 180 days in persistent episodes). Stratified by follow-up length, complete resolution was documented in 18/18 episodes with follow-up of one month or less, 18/22 with follow-up over one month, and 14/17 when follow-up duration was not reported; non-resolution was concentrated in persistent episodes with follow-up beyond one month, so the pooled proportions are not an artifact of short follow-up. Follow-up remained heterogeneous and often incompletely reported, so these proportions estimate neither prognosis nor treatment effect. Excluding the nerve-block series entirely (n = 66) left the longer-follow-up outcome estimates unchanged by construction and lowered the treated proportion to 34/52 (65.4%) vs. 48/66 (72.7%) (Table S8 and Table 5).

**Table 5:** Treatments administered and outcomes by duration category.

Variable	Overall (n = 80)	Prolonged (n = 49)	Persistent (n = 31)
Treatment directed at the aura/attack, n/N (%)	48/66 (72.7)	24/38 (63.2)	24/28 (85.7)
Agents recorded, number of episodes			
Greater occipital nerve block	14	6	8
Valproate	10	5	5
Lamotrigine	6	0	6
Corticosteroids	5	3	2
Triptans	5	3	2
Topiramate	3	0	3
Furosemide	3	1	2
NSAIDs	3	3	0
Verapamil	2	1	1
Magnesium	2	2	0
Acetazolamide	1	0	1
Outcome at last reported assessment (longer/variable follow-up), n/N (%)			
Aura improved	54/58 (93.1)	36/36 (100.0)	18/22 (81.8)
Complete resolution	50/57 (87.7)	35/35 (100.0)	15/22 (68.2)
Outcome at 24 h after greater occipital nerve block (separate series), n/N (%)			
Aura improved	11/14 (78.6)	5/6 (83.3)	6/8 (75.0)
Complete resolution	5/14 (35.7)	4/6 (66.7)	1/8 (12.5)

Treatment and outcome rows are n/N (%) using the number of episodes for which each variable was ascertainable. Agent rows are counts of episodes in which the agent is named in the treatment record for the index episode; several patients received more than one agent, so these counts are not mutually exclusive and do not sum to the number of treated episodes. These are documented therapeutic attempts, not evidence of comparative effectiveness. CGRP, calcitonin gene-related peptide; NSAID, non-steroidal anti-inflammatory drug. Greater occipital nerve block was administered in a single prospective case series using bilateral bupivacaine 0.5%, 2 mL per side. No episode was treated with ketamine or a CGRP-targeted monoclonal antibody. Agent counts reflect explicitly reported treatments and were verified at the episode level. Follow-up duration and ascertainment were heterogeneous, so outcome proportions are available-case estimates over uncontrolled observation periods.

### 3.5. Critical Appraisal Findings

Item-level appraisal showed a consistent profile across designs: what the primary authors set out to describe was usually documented, whereas the items that determine whether a report can be aggregated with others were not. Adverse events were not reported in 27 of 31 case reports, consecutive or complete inclusion could not be confirmed in 14 of 15 case series, and confounding was inadequately described in the 2 studies appraised with the analytical cross-sectional checklist. All item-level judgements, including those for the 13 studies added during revision, are provided in Supplementary Table S2.

## 4. Discussion

### 4.1. Synthesis of Key Findings

This review assembled 80 episodes from 48 reports and identified three consistent descriptive features. First, visual symptoms predominated, but the strong female representation cannot be generalized beyond the published case literature. Second, MRI was common, yet patient-level informative diffusion imaging was available for only 28 of 80 episodes and imaging-based perfusion for 28, limiting inference about imaging phenotypes. Third, complete resolution was reported less often for persistent than for prolonged episodes in the longer-follow-up stratum (15/22 vs. 35/35). This difference is partly expected by construction, because an episode classified as persistent had, by definition, not resolved within 7 days; it is also influenced by unequal and often unreported follow-up and by publication selection, and should therefore not be interpreted as a prognostic difference between the two duration categories.

Earlier narrative syntheses used broader and inconsistent eligibility definitions and did not report a registered search, study flow, item-level appraisal, or available-case denominators [4,83]. Their central observations (predominance of visual aura, female over-representation, heterogeneous imaging, and insufficient therapeutic evidence) are broadly consistent with ours. The present review adds reproducible methods, explicit episode-level eligibility, structured diffusion/ADC and perfusion extraction, and a transparent account of missing data; it does not convert case observations into population estimates.

### 4.2. Pathophysiology and Mechanisms

Cortical spreading depolarization is the leading neurophysiological correlate of typical aura [84,85], but the mechanism sustaining symptoms for days or weeks remains uncertain. The included reports described migrating, fully reversible diffusion abnormalities, elevated or non-restricted ADC, and transient contrast leakage [5,37,38,54], findings compatible with reversible neurovascular or blood–brain barrier disturbance rather than fixed infarction. A magnetoencephalographic study of six patients with persistent visual aura from a single clinic, two of whom described a TV-static phenotype, also reported sustained visual cortex hyperexcitability [86]. None of these observations directly measured spreading depolarization or established causality, and widely varying imaging intervals limit mechanistic interpretation.

### 4.3. Neuroimaging, Differential Diagnosis, and Exclusions

The five diffusion-positive episodes did not share a uniform signature: one showed a non-restricted (vasogenic) pattern [5], and in four the abnormality was classified as compatible with the absence of established infarction only after normalization on repeat imaging, with the acute ADC reduced in three and not reported in one [38,39,53,64]. In one of these, serial imaging documented a reduced ADC on day 2 (0.00054 vs. 0.00071 mm^2^/s contralateral), migration of the abnormality from the occipital to the temporoparietal cortex by day 17, and normalization of all sequences and of the ADC by day 56; the authors considered this course incompatible with a fixed arterial infarct territory [38]. Perfusion reports described hypo- and hyperperfusion and transitions between them, but acquisition methods and timing were too heterogeneous for pooled directional estimates. One included report documented an ADC abnormality with a normal diffusion-weighted image [37], and a pediatric series of children with aura lasting 1–24 h, which included patients with hemiparesis, reported group-level SPECT hypoperfusion in the symptomatic hemisphere on the day of admission and slight hyperperfusion on the following day [87]. A prolonged aphasic aura with delayed left-hemispheric hyperperfusion on SPECT on day 5, which had normalized by 6 weeks, has also been reported in a patient with genetically confirmed cerebral autosomal dominant arteriopathy with subcortical infarcts and leukoencephalopathy (CADASIL) [88]; this report was excluded from the episode-level synthesis because the aura was attributable to an underlying monogenic cerebral small-vessel disease. These observations highlight the importance of ADC maps, acquisition timing, vascular imaging, and follow-up; they do not provide a rule for distinguishing aura from acute ischemia at first presentation.

Two features of the diffusion-positive episodes follow from how they were selected and should not be read as findings. Under the amended eligibility rule, a diffusion abnormality was admissible only after established infarction had been excluded and complete radiologic normalization had been documented; the presence of such episodes in this dataset therefore demonstrates only that such reports have been published, not that a positive diffusion signal is compatible with aura. Likewise, the distinction between non-restricted (vasogenic) and restricted (cytotoxic-appearing) patterns is a classification by ADC, not an independent observation. The observation of practical value is a different one: in 4 of the 5 diffusion-positive episodes, the abnormality could be classified as compatible with the absence of established infarction only on repeat imaging, so the question could not be settled at the first imaging study. These are descriptions of what the published reports contained, not diagnostic criteria: the review cannot estimate the diagnostic performance of any clinical or imaging feature, and no included report documented a uniform secondary workup. Prolonged focal symptoms therefore continue to require urgent exclusion of ischemic stroke, TIA, RCVS, arterial dissection, seizure, hemiplegic migraine, migrainous infarction, posterior reversible encephalopathy syndrome, inflammatory or prothrombotic disease, and selected genetic or mitochondrial disorders [6,7,8], and normal or reversible findings in published cases must not be used to defer appropriate acute cerebrovascular assessment.

### 4.4. Interpretation of Treatment Effects and Outcomes

The treatments in the included reports were heterogeneous and usually administered without a comparator. Improvement after lamotrigine, valproate, furosemide, corticosteroids, triptans, or greater occipital nerve block cannot be attributed to the intervention because spontaneous remission, publication selection, co-treatment, and variable follow-up are inseparable from the observed outcome. A double-blind, randomized, active-controlled trial outside the episode-level dataset compared intranasal ketamine with intranasal midazolam in 30 patients with migraine with prolonged aura, of whom 18 provided outcome data [89]. Ketamine reduced a composite aura-severity score but not aura duration, and midazolam did not change severity. The trial population differs from that of the present review: 15 of the 18 analyzed patients had motor aura, 14 of whom were classified as having sporadic or familial hemiplegic migraine, and the severity score included motor items. These findings therefore cannot be extrapolated to non-motor prolonged aura; at most, they provide a mechanistic rationale for further study. No episode was treated with a calcitonin gene-related peptide (CGRP)-targeted monoclonal antibody, reflecting the publication dates of most included reports. No agent can be considered preferred on the basis of the current case literature.

### 4.5. Limitations

Several limitations should be acknowledged. First, the evidence is a publication-selected set of case reports and small series rather than a representative cohort. The proportions reported here therefore describe the published record and are not population estimates. Second, episodes are not independent: they were clustered within studies of unequal size, and one patient contributed two episodes. Third, imaging and outcome data were heterogeneous and incomplete. Patient-level informative diffusion imaging was available in 28 of 80 episodes and imaging-based perfusion in 28, follow-up and outcome ascertainment varied, and available-case denominators ranged from 57 to 80. In addition, whether imaging was actually acquired while aura symptoms were present could not be established for 26 of 80 episodes (32.5%), including all 14 episodes of the nerve-block series; for these, imaging findings cannot be assigned to the symptomatic phase, and a normal perfusion or diffusion study may reflect acquisition after a transient physiological change had resolved. Inferences about the imaging correlates of the symptomatic phase rest, in effect, on the 53 episodes with documented symptomatic-phase imaging. Fourth, a lifetime history of motor aura was explicitly addressed in only 12 episodes, so the exclusion of hemiplegic migraine rests in most instances on the absence of reported weakness rather than on its documented absence. Fifth, the overall female predominance cannot be judged against expectation because population estimates of the sex ratio in migraine with aura range from approximately 1:2 male to female to near parity [90,91]. The departure that does not depend on that choice is confined to the persistent subgroup, in which 30 of 31 episodes occurred in women compared to half of 4 cases in a consecutive clinic series of 477 patients [92].

Finally, several search-related limitations remain despite the October 2026 update. The original database strings paired aura terms only with duration terms and used no controlled vocabulary; the update search added Medical Subject Headings in PubMed, an explicit index-term component in Scopus, broader synonym blocks, and forward citation tracking. Embase was not searched in either round because no institutional access was available. Reliance on English-language search terms may have missed reports indexed in regional databases. Google Scholar output is unstable over time: the August 2026 runs were screened on screen with a dated run log recording query, result counts, and every candidate record, but the result pages themselves were not archived, and the eight records unique to the August database update were not individually itemized; the October 2026 Google Scholar runs were archived in full. At least eight of 21 conference abstracts excluded for lacking a full report appeared to contain potentially eligible data, so their exclusion may have removed eligible episodes.

## 5. Conclusions

In this review, published episodes of prolonged and persistent non-motor migraine aura without reported established infarction were imaged frequently but described incompletely: patient-level informative diffusion imaging was available for a minority of episodes, ADC findings were rarely reported, and imaging during ongoing symptoms could often not be established. Because these are publication-selected case observations without comparators, they cannot support estimates of frequency, prognosis, or treatment effect. As a reporting proposal arising from these gaps, rather than a conclusion from the data, we suggest that future reports state the timing of each imaging study relative to symptom onset and resolution, report diffusion-weighted images together with ADC findings, and specify the duration of follow-up.

## Figures and Tables

**Table 1:** Eligibility criteria for individual aura episodes.

Domain	Inclusion Criteria	Exclusion Criteria
Population	Patients of any age and either sex with an established or retrospectively adjudicable diagnosis of migraine with aura (ICHD-3 1.2, including the minimum of two attacks of criterion A). No restriction on comorbidity, country, or care setting.	None applied on demographic grounds.
Condition— aura phenotype	At least one non-motor aura symptom in the index episode: visual, sensory, speech–language, or brainstem.	Motor weakness documented in the index episode. Familial or sporadic hemiplegic migraine. Visual snow syndrome. Retinal migraine. Phenomena not classified as aura in ICHD-3 (for example, olfactory hallucination and metamorphopsia).
Condition— duration	The qualifying non-motor symptom lasted more than 60 min in the index episode. Prolonged: >60 min but <7 days. Persistent: ≥7 days.	All reported episodes lasting 60 min or less. Aura status, in which brief attacks recur with aura-free intervals, unless a single episode itself exceeded 60 min.
Condition— neuroimaging	Neuroimaging temporally related to the index episode was required, and established cerebral infarction had to be absent. Transient diffusion abnormality without radiologically established infarction was permitted (see Registration Amendments).	No neuroimaging reported for the index episode, or imaging obtained only as interictal or diagnostic workup. Radiologically established cerebral or migrainous infarction.
Context	Any clinical setting and any calendar period; no publication year restriction. Language was not applied as an exclusion criterion at the full-text stage: reports in any language were assessed where an English abstract or a translation permitted adjudication.	Aura-like symptoms attributable to another established neurological disorder (arteriovenous malformation, MELAS, Sturge–Weber syndrome, epilepsy, PRES, reversible cerebral vasoconstriction syndrome, arterial dissection, tumor, and similar).
Data	Data reported at the level of the individual patient, sufficient to extract age, sex, aura symptom type, duration, imaging, and outcome for the index episode.	Group-level reporting only. Patients already represented by an included report (duplicate cohort or duplicate publication).
Study designs	Case reports, case series, and observational cohort studies with a complete published report or article.	Reviews, editorials, guidelines, conference-only abstracts or posters, and reports without a complete published text. Preclinical, mechanistic, provocation, interventional, and group comparison imaging studies.
Outcomes (descriptive only)	Clinical phenotype and aura symptom type; aura duration and duration category; neuroimaging findings, including diffusion, apparent diffusion coefficient, and perfusion; treatments administered; aura improvement and complete resolution at last reported assessment.	No comparative effectiveness outcome was sought, and no between-group hypothesis test was planned.

ICHD-3, International Classification of Headache Disorders, third edition; MELAS, mitochondrial encephalomyopathy, lactic acidosis, and stroke-like episodes; PRES, posterior reversible encephalopathy syndrome. Reports not meeting more than one criterion were assigned a single exclusion reason using a pre-specified hierarchy.

**Table 2:** Characteristics of the included studies.

Author (Year)	n	Age (Years)	F/M	Type of Aura	Duration of Aura
Angermaier A (2014) [36]	1	24	0/1	Visual and sensory and speech	1 d
Belvis R (2010) [37]	1	41	1/0	Visual and sensory	9 d
Bereczki D (2008) [38]	1	58	0/1	Visual	15 d
Bormann T (2017) [39]	1	65	1/0	Visual	17 d
Bruen R (2013) [40]	1	14	1/0	Visual	2 mo
Burns (2017) [41]	1	25	1/0	Sensory and speech	29 h
Chen WT (2001) [42]	2	45, 24	2/0	Visual	3 mo; 3 yr
Cuadrado ML (2017) [43]	14	27–61	12/2	Sensory; visual; visual and sensory	7 h; 2.42 h; 3.5 h; 2.25 h; 4 d; 11.5 h; …
de Almeida RF (2009) [44]	1	11	1/0	Visual	7 d
Evans RW (2000) [45]	1	33	1/0	Sensory	11 d
García Cruz A (2026) [46]	1	29	1/0	Sensory and speech	NR
Gonzalez-Martinez A (2021) [47]	2	22, 49	0/2	Speech	NR; NR
Goodwin D (2011) [48]	1	27	1/0	Visual	4 h
Grangeon L (2016) [49]	1	30	1/0	Visual and speech	4 h
Hayashi K (2024) [50]	1	46	0/1	Visual	3 h
Jager HR (2005) [51]	2	27, 33	2/0	Visual	NR; NR
Kaltseis K (2021) [52]	1	16	1/0	Visual and sensory and speech	27 mo
Kulesh AA (2025) [53]	2	24, 33	1/1	Visual and sensory and speech	20 h; 6.5 h
Lanfranconi S (2009) [54]	1	67	1/0	Visual and speech	4 d
Lindner A (1996) [55]	1	41	0/1	Visual and sensory	24 h
Luda E (1991) [56]	1	65	1/0	Visual	1 yr
Mayà-Casalprim G (2020) [57]	1	80	0/1	Speech	2 d
Miller C (2012) [58]	1	22	1/0	Speech	NR
Ming X (2010) [59]	1	15	1/0	Sensory	10 h
Mishra NK (2009) [60]	1	40	1/0	Speech	1 d
Miyazato S (2024) [61]	1	16	0/1	Visual	NR
Nair BS (2025) [62]	1	32	1/0	Visual	7 d
Nieuwkamp DJ (2010) [63]	1	28	1/0	Speech	9 h
Parks NE (2014) [64]	1	59	1/0	Visual and sensory and speech	NR
Perino M (2000) [65]	1	26	1/0	Sensory and speech	NR
Razeghinejad MR (2009) [66]	1	25	1/0	Visual	4 mo
Relja G (2005) [67]	1	43	1/0	Visual	7 d
Resnick S (2006) [5]	1	56	0/1	Visual and sensory	18 h
Rothrock JF (1997) [68]	2	61, 53	2/0	Visual and sensory	2 mo; 2 yr
Rozen TD (2000) [69]	1	37	1/0	Visual	5 d
Rozen TD (2003) [70]	1	27	1/0	Visual	6 d
San-Juan OD (2007) [71]	1	28	1/0	Visual	35 d
Scutelnic A (2024) [72]	7	21–78	5/2	Sensory and speech; visual; visual and sensory; visual and sensory and speech; visual and speech	NR; 24 h; NR; 5 h; 3 d; NR; …
Sethi HS (2012) [73]	2	14, 14	2/0	Visual	5 d; 31.5 d
Shah L (2013) [74]	1	22	1/0	Speech	3 d
Slavova N (2020) [75]	1	38	1/0	Visual and sensory and speech	4 h
Thissen S (2014) [76]	2	41, 59	2/0	Visual	3 mo; NR
Viola S (2011) [77]	7	21–41	4/3	Speech; visual and sensory; visual and sensory and speech; visual and speech	8 h; 3 h; 2 h; 7 h; 4 h; 2 h; …
Wakakura M (1992) [78]	1	39	1/0	Visual	NR
Wang YF (2008) [79]	1	56	1/0	Visual	20 yr
Wolf ME (2018) [80]	1	37	0/1	Visual	2 h
Zayat R (2024) [81]	1	57	0/1	Visual and speech	5.5 d
Zouitina Y (2014) [82]	1	36	0/1	Visual and speech	2 d

n, number of eligible episodes; F/M, female/male episodes; NR, not reported; h, hours; d, days; mo, months; yr, years. Durations are reproduced as reported in the source articles. All 48 included reports were assessed from the complete published article or report. Sethi et al. reported two eligible episodes in the same 14-year-old female; consequently, the dataset contains 80 episodes from 79 individuals.

## Data Availability

All data supporting the reported analyses are contained in the article and in Supplementary Tables S1–S9 and Supplementary File S1. No new primary patient data were generated.

## Supplementary Materials

The following supporting information can be downloaded at https://www.mdpi.com/article/10.3390/neurolint18100197/s1. Table S1: Complete search strategies and yields reported according to PRISMA-S; Table S2: Item-level JBI critical appraisal; Table S3: Full-text exclusions (sheets S3a, S3d, and S3e), citation searching (S3b), unretrieved reports (S3c), and PRISMA reconciliation; Table S4: Departures from the registered PROSPERO record; Table S5: Expanded study characteristics; Table S6: De-identified episode-level analysis dataset; Table S7: Re-adjudication decisions and operational rules; Table S8: Sensitivity analyses; Table S9: Follow-up strata and clinical–imaging concordance; File S1: Reproducibility code and README; PRISMA 2020 checklist. References [1,5,9,10,11,12,16,17,18,19,20,21,22,23,24,25,26,27,28,29,30,31,32,33,34,35,36,37,38,39,40,41,42,43,44,45,46,47,48,49,50,51,52,53,54,55,56,57,58,59,60,61,62,63,64,65,66,67,68,69,70,71,72,73,74,75,76,77,78,79,80,81,82] are cited in the Supplementary Materials.

## Abbreviations

The following abbreviations are used in this manuscript:

AC1	first-order agreement coefficient
ADC	apparent diffusion coefficient
ASL	arterial spin labeling
CADASIL	cerebral autosomal dominant arteriopathy with subcortical infarcts and leukoencephalopathy
CGRP	calcitonin gene-related peptide
CI	confidence interval
CoCoPop	Condition, Context, and Population
CT	computed tomography
DWI	diffusion-weighted imaging
EEG	electroencephalography
ICHD-3	International Classification of Headache Disorders, third edition
IQR	interquartile range
JBI	Joanna Briggs Institute
MELAS	mitochondrial encephalomyopathy, lactic acidosis, and stroke-like episodes
MRI	magnetic resonance imaging
MwA	migraine with aura
NIRS	near-infrared spectroscopy
NR	not reported
NSAID	non-steroidal anti-inflammatory drug
PABAK	prevalence-adjusted bias-adjusted kappa
PET	positron emission tomography
PICO	Population, Intervention, Comparator, and Outcome
PRES	posterior reversible encephalopathy syndrome
PRISMA	Preferred Reporting Items for Systematic Reviews and Meta-Analyses
PRISMA-S	PRISMA extension for reporting literature searches
PROSPERO	International Prospective Register of Systematic Reviews
RCVS	reversible cerebral vasoconstriction syndrome
SPECT	single-photon emission computed tomography
TCD	transcranial Doppler
TIA	transient ischemic attack
