# Supplementary material for: Clinical Phenotypes, Neuroimaging, and Outcomes in Prolonged and Persistent Non-Motor Migraine Aura: A Systematic Review and Episode-Level Synthesis
Source: Neurol Int. 2026 Oct 21;18(10):197. doi: 10.3390/neurolint18100197 (PMC13708082; doi:10.3390/neurolint18100197)
Supplement: Supplementary file 1 [file neurolint-18-00197-s001.zip › PRISMA_2020_checklist.pdf]

PRISMA 2020 Checklist

Clinical Phenotypes, Neuroimaging, and Outcomes in Prolonged and Persistent Non-Motor Migraine Aura: A Systematic Review and Episode-Level Synthesis

PROSPERO CRD420261461238. Locations refer to numbered manuscript sections and named supplementary files.

Section and Topic	Item #	Checklist item	Location where item is reported
**TITLE**			
**Title**	1	Identify the report as a systematic review.	Title
**ABSTRACT**			
**Abstract**	2	See the PRISMA 2020 for Abstracts checklist.	Abstract
**INTRODUCTION**			
**Rationale**	3	Describe the rationale for the review in the context of existing knowledge.	Section 1
**Objectives**	4	Provide an explicit statement of the objective(s) or question(s) the review addresses.	Section 1
**METHODS**			
**Eligibility criteria**	5	Specify the inclusion and exclusion criteria for the review and how studies were grouped for the syntheses.	Sections 2.2, 2.7 and 2.8; Table 1
**Information sources**	6	Specify all databases, registers, websites, organisations, reference lists and other sources searched or consulted to identify studies. Specify the date when each source was last searched or consulted.	Section 2.3; Supplementary Table S1 (original searches, the update search of 5 October 2026, and forward citation tracking)
**Search strategy**	7	Present the full search strategies for all databases, registers and websites, including any filters and limits used.	Section 2.3; Supplementary Table S1 (original searches, the update search of 5 October 2026, and forward citation tracking)

Section and Topic	Item #	Checklist item	Location where item is reported
**Selection process**	8	Specify the methods used to decide whether a study met the inclusion criteria of the review, including how many reviewers screened each record and each report retrieved, whether they worked independently, and if applicable, details of automation tools used in the process.	Section 2.4; Supplementary Tables S4 and S7. Title and abstract screening of the original searches was performed by a single reviewer, as registered; the database branch was first triaged by a rule-based (non-artificial-intelligence) keyword filter, and at revision all keyword-excluded records were re-screened individually and independently by two reviewers (agreement 98.4%, 299/304; PABAK 0.967), recovering one eligible report. The Google Scholar branch was screened manually, record by record, with a written log for each run. In the original review, full texts were independently assessed by two reviewers without automation tools. In the October 2026 update, title/abstract screening and full-text assessment were performed by one reviewer with the aid of a generative artificial-intelligence tool (disclosed in Section 2.4) and verified on a blinded stratified sample of 49 records by the second reviewer (agreement 87.8%); this is a documented departure from the registered dual full-text assessment (Supplementary Table S4). Every previously included episode was re-adjudicated against the registered criteria (Supplementary Table S7).
**Data collection process**	9	Specify the methods used to collect data from reports, including how many reviewers collected data from each report, whether they worked independently, any processes for obtaining or confirming data from study investigators, and if applicable, details of automation tools used in the process.	Section 2.5. Data were extracted by one reviewer and checked against source reports by a second reviewer; disagreements were resolved through discussion and consensus. Study investigators were not contacted for additional data.
**Data items**	10a	List and define all outcomes for which data were sought. Specify whether all results that were compatible with each outcome domain in each study were sought, and if not, the methods used to decide which results to collect.	Section 2.5
	10b	List and define all other variables for which data were sought. Describe any assumptions made about any missing or unclear information.	Section 2.5; Supplementary Table S6

Section and Topic	Item #	Checklist item	Location where item is reported
**Study risk of bias assessment**	11	Specify the methods used to assess risk of bias in the included studies, including details of the tool(s) used, how many reviewers assessed each study and whether they worked independently, and if applicable, details of automation tools used in the process.	Section 2.6; Supplementary Table S2. One reviewer performed the item-level JBI appraisal and a second reviewer checked each judgement; disagreements were resolved through discussion and consensus. For the 13 studies added at revision, draft appraisals were prepared with artificial-intelligence assistance and each judgement was reviewed and confirmed by the authors, as stated in Supplementary Table S2. No summary score or universal category was assigned.
**Effect measures**	12	Specify for each outcome the effect measure(s) used in the synthesis or presentation of results.	Section 2.7. No comparative effect measure was used; results are descriptive counts, proportions, medians, interquartile ranges, and ranges with explicit available-case denominators.
**Synthesis methods**	13a	Describe the processes used to decide which studies were eligible for each synthesis.	Section 2.7
	13b	Describe any methods required to prepare the data for presentation or synthesis, such as handling of missing summary statistics, or data conversions.	Section 2.7
	13c	Describe any methods used to tabulate or visually display results of individual studies and syntheses.	Section 2.7; Tables 2–5; Figure 2
	13d	Describe any methods used to synthesize results and provide a rationale for the choice(s).	Section 2.7. Meta-analysis was not performed; the rationale is provided.
	13e	Describe any methods used to explore possible causes of heterogeneity among study results.	Not applicable — no statistical synthesis was performed, so statistical heterogeneity could not be quantified (Section 2.7).
	13f	Describe any sensitivity analyses conducted to assess robustness of the synthesized results.	Section 2.7; twelve prespecified and post hoc sensitivity scenarios reported in Sections 3.3–3.4 and Supplementary Table S8
**Reporting bias assessment**	14	Describe any methods used to assess risk of bias due to missing results in a synthesis.	Section 2.7 — formal assessment was not undertaken because protocols and prespecified outcomes were unavailable for most primary reports, and the descriptive case-based evidence did not permit a reliable assessment of selective non-reporting. Publication and reporting selection are discussed in Section 4.5.

Section and Topic	Item #	Checklist item	Location where item is reported
**Certainty assessment**	15	Describe any methods used to assess certainty (or confidence) in the body of evidence for an outcome.	Section 2.7 — not undertaken because the uncontrolled case-based evidence provided no estimable comparative effect. See Sections 3.5 and 4.5.
**RESULTS**			
**Study selection**	16a	Describe the results of the search and selection process, from the number of records identified in the search to the number of studies included in the review, ideally using a flow diagram.	Section 3.1; Figure 1
	16b	Cite studies that might appear to meet the inclusion criteria, but which were excluded, and explain why they were excluded.	Section 3.1; Supplementary Table S3
**Study characteristics**	17	Cite each included study and present its characteristics.	Section 3.1; Table 2
**Risk of bias in studies**	18	Present assessments of risk of bias for each included study.	Section 3.5; Supplementary Table S2
**Results of individual studies**	19	For all outcomes, present, for each study: (a) summary statistics for each group and (b) an effect estimate and its precision, ideally using structured tables or plots.	Tables 2–5 and Supplementary Tables S5, S6, and S9. Individual-study and episode-level results are presented; no comparative effect estimate was applicable.
**Results of syntheses**	20a	For each synthesis, briefly summarize the characteristics and risk of bias among contributing studies.	Sections 3.1, 3.5, 4.5
	20b	Present results of all statistical syntheses conducted.	Sections 3.2–3.4; Tables 3–5; Figure 2
	20c	Present results of all investigations of possible causes of heterogeneity among study results.	*Not applicable — see item 13e.*
	20d	Present results of all sensitivity analyses conducted to assess the robustness of the synthesized results.	Sections 3.3 and 3.4; Supplementary Table S8 (twelve sensitivity scenarios) and Supplementary Table S9 (outcomes stratified by follow-up length).
**Reporting biases**	21	Present assessments of risk of bias due to missing results for each synthesis assessed.	Not applicable — see item 14. Discussed narratively in Section 4.5.
**Certainty of evidence**	22	Present assessments of certainty (or confidence) in the body of evidence for each outcome assessed.	Not performed — see item 15. Section 4.5.
**DISCUSSION**			
**Discussion**	23a	Provide a general interpretation of the results in the context of other evidence.	Sections 4.1–4.4
	23b	Discuss any limitations of the evidence included in the review.	Section 4.5
	23c	Discuss any limitations of the review processes used.	Sections 2.3, 4.5 and Supplementary Table S4
	23d	Discuss implications of the results for practice, policy, and future research.	Sections 4.5 and 5
**OTHER INFORMATION**			

Section and Topic	Item #	Checklist item	Location where item is reported
**Registration and protocol**	24a	Provide registration information for the review, including register name and registration number, or state that the review was not registered.	Section 2.1. PROSPERO CRD420261461238; version 1.0 published 26 July 2026, version 2.0 published 28 August 2026, and version 2.1 published 1 September 2026.
	24b	Indicate where the review protocol can be accessed, or state that a protocol was not prepared.	Section 2.1. No separate full protocol was prepared.
	24c	Describe and explain any amendments to information provided at registration or in the protocol.	Sections 2.8 and 4.5; Supplementary Table S4. All registered-versus-conducted differences, including those arising during the revision, are identified, dated, and explained; the registration itself was not amended after peer review began.
**Support**	25	Describe sources of financial or non-financial support for the review, and the role of the funders or sponsors in the review.	Funding<br>*Science Committee of the Ministry of Science and Higher Education of the Republic of Kazakhstan, Grant No. AP26197925. The funder had no role in the review.*
**Competing interests**	26	Declare any competing interests of review authors.	Conflicts of Interest<br>*The authors declare no conflicts of interest.*
**Availability of data, code and other materials**	27	Report which of the following are publicly available and where they can be found: template data collection forms; data extracted from included studies; data used for all analyses; analytic code; any other materials used in the review.	Data Availability Statement; Supplementary Tables S1–S9 and Supplementary File S1. Supplementary Table S6 contains the frozen de-identified episode-level dataset; Supplementary File S1 contains the reproducibility code and a README.
