# Supplementary material for: Clinical Phenotypes, Neuroimaging, and Outcomes in Prolonged and Persistent Non-Motor Migraine Aura: A Systematic Review and Episode-Level Synthesis
Source: Neurol Int. 2026 Oct 21;18(10):197. doi: 10.3390/neurolint18100197 (PMC13708082; doi:10.3390/neurolint18100197)
Supplement: Supplementary file 1 [file neurolint-18-00197-s001.zip › Supplementary_Table_S1_Search_strategy.pdf]

Supplementary Table S1. Complete search strategies and yields

Reported in accordance with PRISMA-S. Rows 1–17 reproduce the original searches as submitted. Rows 18–24 document the update search executed in response to peer review. All sources were searched from inception with no restriction by publication year; language was not an eligibility filter at any stage.

Google Scholar archiving. The July 2026 preliminary search exported 31 records. The August 2026 runs (rows 4–14) were screened on screen with a dated run log recording each query, its displayed result count, the screening depth, and every full-text candidate; the result pages themselves were not archived, which is acknowledged in Section 4.5 (Limitations). The October 2026 runs (rows 21–22) were archived in full: raw result pages were saved as text and parsed by script.

#	Source	Platform / host	Fields searched	Date of search	Search string or procedure	Results returned	Records screened	Notes
1	PubMed	NCBI	All Fields (see note)	23 August 2026	(“migraine with aura” OR “migraine aura” OR “migrainous aura” OR “typical aura without headache” OR “aura without headache” OR “acephalgic migraine”) AND (prolong* OR persist* OR “persistent aura without infarction” OR “persistent migraine aura” OR “prolonged migraine aura” OR “persistent migrainous aura” OR “prolonged migrainous aura”)	466	466	All records exported and screened. Field descriptor recorded as All Fields (re-execution note as in the submitted version).
2	Scopus	Elsevier	TITLE-ABS-KEY	23 August 2026	TITLE-ABS-KEY ((“migraine with aura” OR “migraine aura” OR “migrainous aura” OR “typical aura without headache” OR “aura without headache” OR “acephalgic migraine”) AND (prolong* OR persist* OR “persistent aura without infarction” OR “persistent migraine aura” OR “prolonged migraine aura” OR “persistent migrainous aura” OR “prolonged migrainous aura”))	725	725	All records exported and screened (RIS).
3	Google Scholar, preliminary search	Google	Full text, relevance-ranked	26 July 2026	Registered string (as row 1)	approx. 17,400	31	First 31 relevance-ranked records exported (pragmatic truncation);

#	Source	Platform / host	Fields searched	Date of search	Search string or procedure	Results returned	Records screened	Notes
								10 unique to Google Scholar.
4	Google Scholar, run A-1	Google	Full text, relevance -ranked	24 August 2026	“persistent aura without infarction”	approx. 431	400	Fixed depth (pages 1-40); not exhaustive; count fluctuated 431-437.
5	Google Scholar, run A-2	Google	Title only	23 August 2026	allintitle: “persistent aura without infarction”	9	9	Exhaustive.
6	Google Scholar, run A-3	Google	Title only	23 August 2026	allintitle: “prolonged migraine aura”	25	25	Exhaustive.
7	Google Scholar, run A-4	Google	Title only	23 August 2026	allintitle: “persistent migraine aura”	20	20	Exhaustive.
8	Google Scholar, run A-5	Google	Title only	23 August 2026	allintitle: “prolonged migrainous aura”	3	3	Exhaustive.
9	Google Scholar, run A-6	Google	Title only	23 August 2026	allintitle: “persistent migrainous aura”	2	2	Exhaustive.
10	Google Scholar, run A-7	Google	Title only	23 August 2026	allintitle: “persistent aura”	37	37	Exhaustive.
11	Google Scholar, run A-8	Google	Title only	23 August 2026	allintitle: “prolonged aura”	50	50	Exhaustive; displayed count resolved to 50 on the final page.
12	Google Scholar, run B	Google	Full text, relevance -ranked	24 August 2026	“prolonged migraine aura” OR “prolonged migrainous aura”	approx. 457	150	Fixed depth (pages 1-15).
13	Google Scholar, run C	Google	Full text, relevance -ranked	24 August 2026	“persistent migraine aura” OR “persistent migrainous aura”	approx. 551	150	Fixed depth (pages 1-15).
14	Google Scholar, run D	Google	Full text, relevance -ranked	24 August 2026	(“migraine with aura” OR “migraine aura”) AND (“prolonged aura” OR “persistent aura”)	approx. 2,150	100	Fixed depth (pages 1-10).
15	Backward citation searching	Reference lists of the included studies	-	August 2026	Reference lists of the included studies checked; records not previously identified retained.	23	23	
16	Trial registers	-	-	-	Not searched	0	0	Declared as n = 0 in the

#	Source	Platform / host	Fields searched	Date of search	Search string or procedure	Results returned	Records screened	Notes
								flow diagram.
17	Websites, organisations, grey literature	-	-	-	Not searched as a separate source	0	0	Grey-literature items encountered through Google Scholar adjudicated within runs A-1 to D (Supplementary Table S3d).
	**UPDATE SEARCH (October 2026)**							
18	PubMed (update), lines #1-#7	NCBI	Mixed (MeSH; [tw]; [tiab]; proximity [tiab:~3])	5 October 2026 (PubMed history timestamp 4 October 2026, 23:10 US Eastern Time)	#1 = registered string (row 1). #2 = “Migraine with Aura”[Mesh] OR (migrain*[tw] AND aura*[tw]) OR “aura status”[tiab] OR “acephalgic migraine”[tiab] OR “aura without headache”[tiab] OR “migraine equivalent*”[tiab] OR ”migraine accompaniment*”[tiab] OR “migrainous accompaniment*”[tiab] OR ”complicated migraine”[tiab]. #3 = *prolong*[tw] OR persist*[tw] OR ”long-lasting”[tw] OR ”long lasting”[tw] OR protracted[tw] OR ”sustained aura”[tiab:~3] OR ”continuous aura”[tiab:~3] OR ”status migrainosus”[tw] OR ”aura status”[tw]. #4 = ”stroke mimic*”[tiab] OR “stroke-like”[tiab] OR “Aphasia”[Mesh] OR aphasi*[tiab] OR dysphasi*[tiab] OR	1,788	1,788 exported (MEDLINE); de-duplicated before screening	Search History CSV archived. All 36 PubMed-indexed known items retrieved (recall check).

#	Source	Platform / host	Fields searched	Date of search	Search string or procedure	Results returned	Records screened	Notes
					reversib*[tiab] OR "Diffusion Magnetic Resonance Imaging"[Mesh] OR diffusion[tiab] OR DWI[tiab] OR "Perfusion Imaging"[Mesh] OR perfusion[tiab] OR hyperperfusion[tiab] OR hypoperfusion[tiab] OR "arterial spin label*"*[tiab] OR "Tomography, Emission-Computed, Single-Photon"[Mesh] OR SPECT[tiab]. #5 = #2 AND (#3 OR #4). #6 = #5 NOT (animals[mh] NOT humans[mh]). #7 (FINAL, exported) = #1 OR #6.			
19	Scopus (update), final string S1	Elsevier	TITLE-ABS-KEY and INDEXTERMS	5 October 2026	(TITLE-ABS-KEY (("migraine with aura" OR "migraine aura" OR "migrainous aura" OR "typical aura without headache" OR "aura without headache" OR "acephalgic migraine") AND (prolong* OR persist* OR "persistent aura without infarction" OR "persistent migraine aura" OR "prolonged migraine aura" OR "persistent migrainous aura" OR "prolonged migrainous aura"))) OR (((INDEXTERMS ("migraine with aura") OR TITLE-ABS-KEY ((migrain* W/5 aura*) OR "aura status" OR "acephalgic migraine" OR "aura without headache" OR "migraine equivalent*" OR *"migraine accompaniment*"* OR "migrainous accompaniment*" OR *"complicated migraine" OR "migraine variant*"* OR "migrainous infarct*" OR "migraine-*	3,165	3,165 exported (CSV)	The original Scopus strategy searched title, abstract, and keyword fields; in the revision it was extended with an explicit INDEXTERMS component. Search History screenshot archived; 34 of 34 Scopus-indexed known items retrieved.

#	Source	Platform / host	Fields searched	Date of search	Search string or procedure	Results returned	Records screened	Notes
					associated stroke")) OR (TITLE-ABS-KEY (migrain) AND TITLE-ABS-KEY (aphasi* OR dysphasi* OR alexi* OR hemianop* OR"visual field defect*" OR "visual field loss" OR scotoma* OR photopsi* OR teichopsi* OR "visual disturbance*" OR "visual phenomen*" OR "visual symptom*" OR paresthesi* OR paraesthesi* OR"sensory disturbance*"))) AND (INDEXTERMS ("status migrainosus" OR "aphasia" OR "diffusion weighted imaging" OR "diffusion magnetic resonance imaging" OR "perfusion weighted imaging" OR "perfusion imaging" OR "arterial spin labeling" OR "single photon emission computed tomography" OR "tomography, emission-computed, single-photon") OR TITLE-ABS-KEY (prolong** OR persist* OR "long-lasting" OR "long lasting" OR protracted OR (sustained W/3 aura*) OR (continuous W/3 aura*) OR "status migrainosus" OR "aura status" OR "stroke mimic*" OR "stroke-like" OR aphasi* OR dysphasi* OR reversib* OR diffusion OR dwi OR perfusion OR hyperperfusion OR hypoperfusion OR"arterial spin label*" OR spect OR "single photon emission")))			
20	Scopus forward citation (seeds)	Elsevier	Cited-by of the 39 included-report seeds (REF/EI	5 October 2026	Seed DOI query (sheet Forward_seeds of the search log) selected; View cited by; export all (RIS).	410	410	Forward citation tracking requested by peer review.

#	Source	Platform / host	Fields searched	Date of search	Search string or procedure	Results returned	Records screened	Notes
			D)					
21	Google Scholar, runs G1-G13	Google	Title only (allintitle)	5 October 2026	Thirteen queries, each read to exhaustion; raw result pages archived as text and parsed by script.	301 parsed in total	301	Per-run counts: G1 allintitle: persistent aura = 45; G2 allintitle: prolonged aura = 60; G3 allintitle: “aura status” = 17; G4 allintitle: “status migrainosus” aura = 12; G5 allintitle: persistent visual migraine = 15; G6 allintitle: sustained aura = 2; G7 allintitle: migraine stroke mimic = 8; G8 allintitle: migraine aphasia = 11; G9 allintitle: aura reversible = 19; G10 allintitle: aura diffusion = 14; G11 allintitle: aura perfusion = 36; G12 allintitle: migraine “visual field” = 35; G13 allintitle: “migraine variant” = 27. Reproducibility check: the phrase query of run A-7 re-run by

#	Source	Platform / host	Fields searched	Date of search	Search string or procedure	Results returned	Records screened	Notes
								mistake returned 37, identical to 23 August 2026.
22	Google Scholar cited-by (non-Scopus seeds)	Google	Cited-by links C1-C5	5 October 2026	Cited-by pages of the five seeds not indexed in Scopus (Nair, Hayashi, Ming, Angermaier, Kulesh); pages archived.	13 (C1 0; C2 2; C3 3; C4 7; C5 1)	13	
23	De-duplication and search universe (update)	-	-	5 October 2026	Cross- and within-source de-duplication by PMID, DOI and normalized title (guarded title matching); matched against the July screening log, Supplementary Table S3, the submitted reference list and the seeds.	5,677 input; 3,734 unique	2,957 new records screened	777 unique records had been screened in the original review. The frozen universe (records, strings, hashes) is retained in the audit trail.
24	Citation tracking of the nine new inclusions	Elsevier / Google	Backward (reference lists) and forward (cited-by)	8 October 2026	Reference lists of the nine newly included reports parsed and matched against all prior screening sets (backward); Scopus cited-by of the eight Scopus-indexed new inclusions and Google Scholar cited-by of the remaining report (forward).	Backward: 111 entries parsed, 6 new; forward: 10 records, 4 new	10 assessed	No further eligible report (Supplementary Table S3b).
