# Supplementary material for: Clinical Phenotypes, Neuroimaging, and Outcomes in Prolonged and Persistent Non-Motor Migraine Aura: A Systematic Review and Episode-Level Synthesis
Source: Neurol Int. 2026 Oct 21;18(10):197. doi: 10.3390/neurolint18100197 (PMC13708082; doi:10.3390/neurolint18100197)
Supplement: Supplementary file 1 [file neurolint-18-00197-s001.zip › Supplementary_Table_S2_Critical_appraisal.pdf]

Supplementary Table S2. Design-specific JBI critical appraisal of the 48 included studies (20 studies removed after re-adjudication; 13 studies added — 4 recovered during re-screening and 9 from the update search of 5 October 2026).

Y, yes; U, unclear; N, no; NA, not applicable. Each item was mapped to prespecified extraction fields and rated Yes only when the required information was documented in the source report; items that could not be adjudicated were rated Unclear. JBI checklist responses are presented item by item. No numerical cutoff or low/moderate/high summary category was assigned. Appraisal of the 13 added studies was drafted with the aid of a generative artificial intelligence tool and every item judgement was reviewed and confirmed by the authors (9 October 2026).

Author (year)	Checklist	1	2	3	4	5	6	7	8	9	10
Angermaier A (2014)	JBI Case Reports (8 items)	Y	Y	Y	Y	Y	Y	U	Y		
Belvís R (2010)	JBI Case Reports (8 items)	Y	Y	Y	Y	Y	Y	U	Y		
Bereczki D (2008)	JBI Case Reports (8 items)	Y	Y	Y	Y	Y	Y	U	Y		
Bormann T (2017)	JBI Case Reports (8 items)	Y	Y	Y	Y	Y	Y	U	Y		
Bruen R (2013)	JBI Case Reports (8 items)	Y	Y	Y	Y	Y	Y	U	Y		
Burns (2017)	JBI Case Reports (8 items)	Y	Y	Y	Y	Y	Y	U	Y		
Chen WT (2001)	JBI Case Series (10 items)	Y	Y	Y	U	U	Y	Y	Y	Y	NA
Cuadrado ML (2016)	JBI Case Series (10 items)	Y	Y	Y	Y	Y	Y	Y	Y	Y	NA
de Almeida RF (2009)	JBI Case Reports (8 items)	Y	Y	Y	Y	Y	Y	U	Y		
Evans RW (2000)	JBI Case Reports (8 items)	Y	Y	Y	Y	Y	Y	Y	Y		
García Cruz A (2026)	JBI Case Reports (8 items)	Y	Y	Y	Y	Y	Y	Y	Y		
Gonzalez-Martinez A (2021)	JBI Analytical Cross-Sectional (8 items)	Y	Y	Y	Y	U	U	Y	NA		
Goodwin D (2011)	JBI Case Reports (8 items)	Y	Y	Y	Y	Y	Y	U	Y		
Grangeon L (2016)	JBI Case Reports (8 items)	Y	Y	Y	Y	U	Y	U	Y		
Hayashi K (2024)	JBI Case Reports (8 items)	Y	Y	Y	Y	Y	Y	U	Y		
Jager HR (2005)	JBI Case Series (10 items)	Y	Y	Y	Y	U	Y	Y	Y	Y	NA
Kaltseis K (2021)	JBI Case Reports (8 items)	Y	Y	U	Y	Y	Y	Y	Y		
Kulesh AA (2025)	JBI Case Series (10 items)	N	Y	Y	N	U	Y	Y	Y	Y	NA
Lanfranconi S (2009)	JBI Case Reports (8 items)	Y	Y	Y	Y	Y	Y	U	Y		
Lindner A (1996)	JBI Case Reports (8 items)	Y	Y	Y	Y	Y	Y	U	Y		
Luda E (1991)	JBI Case Reports (8 items)	Y	Y	Y	Y	Y	Y	U	Y		
Mayà-Casalprim G (2020)	JBI Case Reports (8 items)	Y	Y	Y	Y	Y	Y	U	Y		
Miller C (2012)	JBI Case Reports (8 items)	Y	Y	Y	Y	Y	Y	U	Y		
Ming X (2010)	JBI Case Series (10 items)	U	Y	Y	N	U	Y	Y	Y	Y	NA
Mishra NK (2009)	JBI Case Reports (8 items)	Y	Y	Y	Y	U	Y	U	Y		
Miyazato S (2024)	JBI Case Reports (8 items)	Y	Y	Y	Y	Y	Y	U	Y		

Author (year)	Checklist	1	2	3	4	5	6	7	8	9	10
Nair BS (2025)	JBI Case Reports (8 items)	Y	Y	Y	Y	Y	Y	U	Y		
Nieuwkamp DJ (2010)	JBI Case Reports (8 items)	Y	Y	Y	Y	Y	Y	U	Y		
Parks NE (2014)	JBI Case Reports (8 items)	Y	Y	Y	Y	Y	Y	U	Y		
Perino F (2000)	JBI Case Reports (8 items)	Y	Y	Y	Y	Y	Y	U	Y		
Razeghinejad MR (2009)	JBI Case Reports (8 items)	Y	Y	Y	Y	Y	Y	U	Y		
Relja G (2005)	JBI Case Reports (8 items)	Y	Y	Y	Y	Y	Y	U	Y		
Resnick S (2006)	JBI Case Reports (8 items)	Y	Y	Y	Y	Y	Y	U	Y		
Rothrock JF (1997)	JBI Case Series (10 items)	Y	N	Y	N	U	Y	Y	Y	Y	NA
Rozen TD (2000)	JBI Case Series (10 items)	Y	Y	Y	U	U	Y	Y	U	Y	NA
Rozen TD (2003)	JBI Case Series (10 items)	Y	Y	Y	U	U	Y	Y	U	Y	NA
San-Juan OD (2007)	JBI Case Reports (8 items)	Y	Y	Y	Y	Y	Y	U	Y		
Scutelnic A (2024)	JBI Analytical Cross-Sectional (8 items)	Y	Y	Y	Y	U	U	Y	NA		
Sethi HS (2012)	JBI Case Reports (8 items)	Y	Y	Y	Y	Y	Y	Y	Y		
Shah L (2013)	JBI Case Reports (8 items)	Y	Y	Y	Y	Y	Y	U	Y		
Slavova N (2020)	JBI Case Series (10 items)	U	Y	Y	N	U	Y	Y	U	Y	NA
Thissen S (2014)	JBI Case Series (10 items)	Y	Y	Y	U	U	Y	Y	U	Y	NA
Viola S (2011)	JBI Case Series (10 items)	Y	Y	Y	U	U	Y	Y	Y	Y	NA
Wakakura M (1992)	JBI Case Series (10 items)	N	Y	Y	N	U	Y	Y	Y	Y	NA
Wang YF (2008)	JBI Case Series (10 items)	Y	Y	Y	U	U	Y	U	U	Y	NA
Wolf ME (2018)	JBI Case Series (10 items)	Y	Y	Y	U	U	Y	Y	Y	Y	NA
Zayat R (2024)	JBI Case Series (10 items)	Y	Y	Y	N	U	Y	Y	U	Y	NA
Zouitina Y (2014)	JBI Case Reports (8 items)	Y	Y	Y	Y	Y	Y	U	Y		

Checklist items (abbreviated from the standard JBI critical appraisal instruments; the item numbers in the column headings refer to the checklist named in each row).

JBI Checklist for Case Reports (items 1–8): 1, patient demographic characteristics clearly described; 2, patient history clearly described and presented as a timeline; 3, current clinical condition on presentation clearly described; 4, diagnostic tests or assessment methods and their results clearly described; 5, intervention or treatment clearly described; 6, post-intervention clinical condition clearly described; 7, adverse or unanticipated events identified and described; 8, takeaway lessons provided.

JBI Checklist for Case Series (items 1–10): 1, clear criteria for inclusion in the series; 2, condition measured in a standard, reliable way for all participants; 3, valid methods used for identification of the condition; 4, consecutive inclusion of participants; 5, complete inclusion of participants; 6, clear reporting of participant demographics; 7, clear reporting of clinical information; 8, outcomes or follow-up results clearly reported; 9, clear reporting of the presenting site or clinic demographic information; 10, appropriate statistical analysis. Item 10 was rated not applicable for purely descriptive series.

JBI Checklist for Analytical Cross-Sectional Studies (items 1–8): 1, inclusion criteria clearly defined; 2, study subjects and setting described in detail; 3, exposure measured in a valid and reliable way; 4, objective, standard criteria used for measurement of the condition; 5, confounding factors identified; 6, strategies to deal with confounding stated; 7, outcomes measured in a valid and reliable way; 8, appropriate statistical analysis. Item 8 was rated not applicable where the report was used descriptively.
