# Supplementary material for: Clinical Phenotypes, Neuroimaging, and Outcomes in Prolonged and Persistent Non-Motor Migraine Aura: A Systematic Review and Episode-Level Synthesis
Source: Neurol Int. 2026 Oct 21;18(10):197. doi: 10.3390/neurolint18100197 (PMC13708082; doi:10.3390/neurolint18100197)
Supplement: Supplementary file 1 [file neurolint-18-00197-s001.zip › Supplementary_Table_S4_Protocol_departures.pdf]

Supplementary Table S4. Departures from the registered PROSPERO record (CRD420261461238)

Reported in accordance with PRISMA 2020 item 24c. Rows 1-9 reproduce the departures documented at submission (incorporated into PROSPERO version 2.0, 28 August 2026; version 2.1 registered single-reviewer title/abstract screening and dual full-text assessment). Rows 10-18 document the departures made during peer-review revision (October 2026), each with its date. The registry record was not amended after peer review: every post-review change was made after the searches and analyses and could not be prospective; this table is the complete disclosure, and the record will be updated to Published with the article DOI upon acceptance.

№	Item	As registered	As conducted	Direction	Reason and consequence
1	Review title	"Neuroimaging Findings in Prolonged and Persistent Non-Motor Migraine Aura Without Established Cerebral Infarction: A Systematic Review"	Title broadened to name the clinical phenotype and outcome domains explicitly	Wording only	Clinical phenotype, clinical outcome and clinical-imaging concordance were all registered as main outcomes.
2	Diffusion-weighted imaging	Negative DWI required for eligibility	Episodes with transient DWI abnormality admitted where established infarction was excluded and reversibility documented on follow-up imaging	Broadening	The original wording would have excluded reports with a completely resolving diffusion abnormality. The amendment was applied symmetrically to previously excluded reports during revision (DWI-amendment re-screen; Supplementary Table S7, sheet 3).
3	Language of publication	Inclusion restricted to English and Russian	No language filter applied at any stage; no report excluded on linguistic grounds	Broadening	Non-English reports among the included studies were assessed and extracted with the aid of online machine translation (Methods 2.5). The residual limitation is one of search coverage, not of eligibility assessment.
4	Information sources	Google Scholar and PubMed	PubMed, Scopus and Google Scholar; update search of 5 October 2026 added Scopus forward citation	Broadening	Scopus was added before screening began.

№	Item	As registered	As conducted	Direction	Reason and consequence
5	Conference abstracts	Excluded only where they lacked sufficient individual-patient data	All reports available only as conference abstracts, posters or presentation records excluded by publication type	RESTRICTION	21 such records were identified at submission, of which at least eight contained extractable data; the cost is stated in Section 4.5 (Limitations).
6	Unpublished studies	Both published and unpublished studies to be sought	One unpublished case report (Google Scholar) excluded for want of peer review	RESTRICTION	Listed in Supplementary Table S3d.
7	Ocular motor palsy	Not addressed; the record excluded motor weakness but was silent on ophthalmoplegia and ptosis	Episodes with documented ocular motor palsy excluded	RESTRICTION	Not among ICHD-3 aura symptoms. Applied symmetrically at revision: two comparator reports relabelled (Supplementary Table S3a) and two previously included episodes excluded (Klapper; Supplementary Table S7).
8	Critical appraisal instrument	JBI tools specified; case report vs case series threshold not pre-specified	Allocation rule fixed post hoc and applied uniformly	Clarification	The rule moved reports toward the instrument with more demanding items.
9	Data collection process	Extraction by one person, checked by at least one other	Extraction by one reviewer, checked field by field by a second; consensus for disagreements	No deviation	Recorded for completeness. Primary investigators were not contacted.
10	Search strategy (update)	Original strings paired aura terms with duration terms; no controlled vocabulary	Searches rerun on 5 October 2026 with Medical Subject Headings (PubMed), an explicit INDEXTERMS component (Scopus), broader synonym blocks (aura status, status migrainosus, stroke mimic, aphasia, reversible imaging findings) and	Broadening (5 Oct 2026)	In response to peer review. 5,677 records identified, 2,957 new records screened, nine studies added (Figure 1; Supplementary Tables S1 and S3e).

№	Item	As registered	As conducted	Direction	Reason and consequence
			thirteen archived Google Scholar allintitle queries		
11	Citation tracking	Backward citation searching of included studies	Forward citation tracking added (Scopus cited-by for all included reports; Google Scholar cited-by for reports not indexed in Scopus), iterated for the nine new inclusions; backward tracking of the new inclusions	Broadening (completed 8 Oct 2026)	Ten further records identified; none eligible (Supplementary Table S3b).
12	Title/abstract screening of the update	Single reviewer (PROSPERO v2.1)	Single reviewer (Z.A.) as registered, with a generative AI tool drafting provisional assessments; the final decision for every record was made by the reviewer	Process disclosure (6 Oct 2026)	AI assistance was not registered; it is disclosed in Methods 2.4 and in the AI-use declaration. Calibration against the pilot and the dual-screened keyword-triage set is documented in the decision logs.
13	Full-text assessment of the update records	Two independent reviewers (PROSPERO v2.1)	Single reviewer (Z.A.) with AI-assisted drafting of provisional assessments; blinded verification by the second reviewer (A.M., without AI) of a stratified sample of 49 records: all candidate inclusions, all borderline records, and a random sample of exclusions	DEPARTURE (8 Oct 2026)	Necessitated by the revision timeline. Independent-phase agreement 87.8% (43/49; Wilson 95% CI 75.8-94.3%; Cohen's kappa 0.62; PABAK 0.76; Gwet's AC1 0.82); every discordant record resolved by consensus on the source full text (Supplementary Table S7). Signed reviewer declarations are retained in the audit trail.
14	Operational full-text rules	Eligibility criteria as registered (Table 1)	Four operational rules fixed during the update full-text pass and applied to	Clarification with restrictive effect (6-7	Operationalizations of registered criteria, recorded per record with dates (Supplementary

№	Item	As registered	As conducted	Direction	Reason and consequence
			all records: duration above 60 minutes must be explicit (6 Oct); acute confusional migraine excluded (6 Oct); episodes attributed by the source authors to a non-migraine cause excluded, whereas an alternative cause that could merely not be ruled out led to retention with a flag (7 Oct); reports predating clinical computed tomography excluded for want of eligible parenchymal imaging (7 Oct)	Oct 2026)	Table S7); none of the nine included update reports predates 1991.
15	Re-adjudication of included episodes	Not foreseen	Every previously included episode re-adjudicated against the registered criteria in response to peer review; 20 studies excluded, 4 previously excluded reports recovered (Belvis, Bruen, Parks, Kulesh); the largest series recoded to its constituent patients and an episode-selection rule for multi-episode reports fixed	Correction (2-4 Oct 2026)	Each decision reported item by item in Supplementary Table S7; study flow updated (Figure 1; Supplementary Table S3).
16	Keyword triage verification	Keyword-based triage of database records (rule-based, non-AI)	All keyword-excluded records re-screened individually and independently by	Verification (4 Oct 2026)	Agreement 98.4% (299/304); the eight records unique to the August 2026 update were not individually itemized

№	Item	As registered	As conducted	Direction	Reason and consequence
			two reviewers; seven proceeded to full text, one (Parks) proved eligible		(Limitations).
17	Sensitivity analyses	Two prespecified scenarios	Two prespecified scenarios reported, plus ten scenarios specified during revision (including exclusion of the 26 flagged episodes and leave-one-study-out)	Addition, labelled post hoc (Oct 2026)	All reported in Supplementary Table S8; headline estimates stable.
18	Outcome analysis strata	Outcomes at last reported assessment	Two prespecified strata: longer/variable follow-up, and the nerve-block series with a fixed 24-hour assessment reported separately; follow-up duration distribution and outcome stratified by follow-up length added	Addition (8 Oct 2026)	In response to peer review (Table 5; Supplementary Table S9).
