# Supplementary material for: Clinical Phenotypes, Neuroimaging, and Outcomes in Prolonged and Persistent Non-Motor Migraine Aura: A Systematic Review and Episode-Level Synthesis
Source: Neurol Int. 2026 Oct 21;18(10):197. doi: 10.3390/neurolint18100197 (PMC13708082; doi:10.3390/neurolint18100197)
Supplement: Supplementary file 1 [file neurolint-18-00197-s001.zip › Supplementary_Table_S5_Study_characteristics.pdf]

Supplementary Table S5. Characteristics of the 48 included studies, full version

Abbreviations as in the submitted version. Durations are as reported in the source article. “None given” means the source explicitly records no treatment; “Not reported” means treatment was not described. Outcome denominators are available-case (episodes with the outcome reported as present or absent); episode counts reflect the re-adjudicated dataset, so for three partially retained series (Cuadrado, Wang, González-Martínez) n and outcome counts differ from the submitted version. Outcomes of the 24-hour nerve-block series are analyzed separately in the main text. Reference numbers follow the reference list of the revised manuscript, in which references are numbered in order of first appearance.

Author (year)	n	Age (years)	F/M	Type of aura	Duration of aura	Investigations	Treatment and outcome
Angermaier A (2014) [36]	1	24	0/1	Visual and sensory and speech	1 d	CT perfusion during episode: left hemispheric hypoperfusion crossing	
vascular territories; MRI day 4 normal	Analgesic, antiemetic; improved 1/1, CR 1/1						
Belvis R (2010) [37]	1	41	1/0	Visual and sensory	9 d	MRI during episode: isolated ADC abnormality, left occipital, with normal T1/T2/FLAIR/DWI; MR angiography, carotid ultrasound, transesophageal echocardiography, Holter, thrombophilia screen normal; MRI day 8 normalized	Rizatriptan, ibuprofen; improved 1/1, CR 1/1
Bereczki D (2008) [38]	1	58	0/1	Visual	15 d	CT/MRI: DWI+ with reduced ADC, normalized, cortical edema	Not reported; improved 1/1, CR 1/1
Bormann T (2017) [39]	1	65	1/0	Visual	17 d	MRI: DWI+, reversible	Corticosteroid, flunarizine; improved 1/1, CR 1/1
Bruen R (2013)	1	14	1/0	Visual	2 mo	Neurologic and ophthalmologic	None given; improved 1/1,

Author (year)	n	Age (years)	F/M	Type of aura	Duration of aura	Investigations	Treatment and outcome
[40]						examination, Goldmann fields, electroretinogram, MRI: all normal	CR 1/1
Burns (2017) [41]	1	25	1/0	Sensory and speech	29 h	MRI: hypoperfusion, hyperperfusion	None given; improved 1/1, CR 1/1
Chen WT (2001) [42]	2	45, 24	2/0	Visual	3 mo; 3 yr	MRI/SPECT: hypoperfusion	Flunarizine, lamotrigine, propranolol; improved 2/2, CR 2/2
Cuadrado ML (2016) [43]	14	27–61	12/2	Sensory; Visual; Visual and sensory	7 h; 2.42 h; 3.5 h; 2.25 h; 4 d; 11.5 h; …	MRI/MRA: no infarction	GON block; improved 11/14, CR 5/14
de Almeida RF (2009) [44]	1	11	1/0	Visual	7 d	MRI: no infarction	Furosemide; improved 1/1, CR 1/1
Evans RW (2000) [45]	1	33	1/0	Sensory	11 d	MRI/MRA: no infarction	Aspirin, furosemide, verapamil; improved 1/1, CR 1/1
García Cruz A (2026) [46]	1	29	1/0	Sensory and speech	NR	Non-contrast CT (ASPECTS 10), CT angiography without occlusion, CT perfusion non-interpretable (motion); delayed MRI normal	Intravenous tenecteplase; improved 1/1, CR 1/1
Gonzalez-Martinez A (2021) [47]	2	22, 49	0/2	Speech	NR; NR	CT: hypoperfusion	None given; improved 2/2, CR 2/2
Goodwin D (2011)	1	27	1/0	Visual	4 h	MRI: no infarction	None given; improved 1/1,

Author (year)	n	Age (years)	F/M	Type of aura	Duration of aura	Investigations	Treatment and outcome
[48]							CR 1/1
Grangeon L (2016) [49]	1	30	1/0	Visual and speech	4 h	MRI 1 h after onset: DWI normal; ASL large left cortical hypoperfusion not matching an arterial territory	Not reported; improved 1/1, CR 1/1
Hayashi K (2024) [50]	1	46	0/1	Visual	3 h	T2-FLAIR MRI normal; ASL: right hemispheric hypoperfusion; MRA	
normal; CSF opening pressure 30.5 cmH2O	IV diazepam, sumatriptan; improved 1/1, CR 1/1						
Jager HR (2005) [51]	2	27, 33	2/0	Visual	NR; NR	MRI: no infarction	None given; improved 0/2, CR 0/2
Kaltseis K (2021) [52]	1	16	1/0	Visual and sensory and speech	27 mo	MRI/MRA: no infarction	Corticosteroid, dronabinol, flunarizine, lamotrigine, levetiracetam,
pregabalin, topiramate, valproate, zonisamide; improved 1/1, CR 1/1							
Kulesh AA (2025) [53]	2	24, 33	1/1	Visual and sensory and speech	20 h; 6.5 h	CT perfusion and MRI with DWI per case; transient cortical signal changes without infarction; follow-up MRI normalized	Per case (incl. intravenous thrombolysis in one); improved 2/2, CR 2/2

Author (year)	n	Age (years)	F/M	Type of aura	Duration of aura	Investigations	Treatment and outcome
Lanfranconi S (2009) [54]	1	67	1/0	Visual and speech	4 d	CT/Doppler ultrasonography/EEG/ MRI/…: hyperperfusion	Aspirin, flunarizine; improved 1/1, CR 1/1
Lindner A (1996) [55]	1	41	0/1	Visual and sensory	24 h	EEG during symptoms: occipital epileptic discharges; MRI: right visual-cortex low perfusion with meningeal hyperemia, reversible	Antiepileptic drugs, calcium-channel blocker; improved 1/1, CR 1/1
Luda E (1991) [56]	1	65	1/0	Visual	1 yr	CT/SPECT: hypoperfusion	Flunarizine, nimodipine; improved 0/1, CR 0/1
Mayà-Casalprim G (2020) [57]	1	80	0/1	Speech	2 d	CT/MRI during episode: no new findings; EEG left theta slowing	Propranolol; improved 1/1, CR 1/1
Miller C (2012) [58]	1	22	1/0	Speech	NR	CT perfusion and SWI during symptoms: regional perfusion abnormality; MRI/DWI without infarction; repeat imaging	Empiric antiviral/antibacterial until diagnosis; improved 1/1, CR 1/1
Ming X (2010) [59]	1	15	1/0	Sensory	10 h	MRI/MRA during episode: normal, no infarction	Valproate, verapamil; improved 1/1, CR 1/1
Mishra NK (2009) [60]	1	40	1/0	Speech	1 d	CT with perfusion and CTA at 1 h: no ischaemia; MRI with DWI and	
perfusion next day: no change; EEG slowing	Not reported; improved 1/1, CR 1/1						
Miyazato S (2024) [61]	1	16	0/1	Visual	NR	CT, contrast CT, serial MRI with DWI and ASL, MRA: ASL hypoperfusion	NSAID, lomerizine; improved 1/1, CR 1/1

Author (year)	n	Age (years)	F/M	Type of aura	Duration of aura	Investigations	Treatment and outcome
						alternating sides; ASL normalized day 2; distal left PCA narrowing only slightly improved; no later imaging	
Nair BS (2025) [62]	1	32	1/0	Visual	7 d	ASL: hypoperfusion	Lasmiditan, propranolol; improved 1/1, CR 0/0
Nieuwkamp DJ (2010) [63]	1	28	1/0	Speech	9 h	CT: hypoperfusion	Not reported; improved 1/1, CR 1/1
Parks NE (2014) [64]	1	59	1/0	Visual and sensory and speech	NR	Serial MRI: apparent cortical laminar necrosis not obeying a vascular territory; follow-up MRI at 18 months with resolution	Aspirin; improved 1/1, CR 0/0
Perino F (2000) [65]	1	26	1/0	Sensory and speech	>3 h (exact total not reported)	CT during attack and EEG normal; CT and MRI at 1 week normal	Ketoprofen, then subcutaneous sumatriptan; improved 1/1, CR 1/1
Razeghinejad MR (2009) [66]	1	25	1/0	Visual	4 mo	MRI/MRA/Transcranial Doppler ultrasonography: no infarction	Propranolol, valproate; improved 1/1, CR 1/1
Relja G (2005) [67]	1	43	1/0	Visual	7 d	CT/MRI/SPECT/Perfusion-weighted MRI: hypoperfusion	Lamotrigine, triptan; improved 1/1, CR 0/1
Resnick S (2006) [5]	1	56	0/1	Visual and sensory	18 h	MRI: DWI+ with elevated ADC, cortical edema	Valproate; improved 1/1, CR 1/1
Rothrock JF (1997) [68]	2	61, 53	2/0	Visual and sensory	2 mo; 2 yr	MRI: no infarction	Amitriptyline, propranolol, valproate; improved 2/2, CR 2/2

Author (year)	n	Age (years)	F/M	Type of aura	Duration of aura	Investigations	Treatment and outcome
Rozen TD (2000) [69]	1	37	1/0	Visual	5 d	MRI/MRA/MRV: no infarction	Corticosteroid, furosemide, magnesium; improved 1/1, CR 1/1
Rozen TD (2003) [70]	1	27	1/0	Visual	6 d	MRI/MRA/MRV: no infarction	Magnesium, prochlorperazine; improved 1/1, CR 1/1
San-Juan OD (2007) [71]	1	28	1/0	Visual	35 d	CT/MRI: no infarction	Nimodipine, topiramate, triptan; improved 1/1, CR 1/1
Scutelnic A (2024) [72]	7	21–78	5/2	Sensory and speech; Visual; Visual and sensory; Visual and sensory and speech; Visual and speech	NR; 24 h; NR; 5 h; 3 d; NR; …	Acute multimodal MRI without ischemic injury (study-level inclusion criterion); sequences and patient-level diffusion findings not reported	Treatment and longitudinal outcomes not reported at episode
level; outcome not reported							
Sethi HS (2012) [73]	2	14, 14	2/0	Visual	5 d; 31.5 d	MRI brain and orbits with contrast (both episodes): unremarkable;	
MRA	None						

Author (year)	n	Age (years)	F/M	Type of aura	Duration of aura	Investigations	Treatment and outcome
neck, TTE, EEG, TCD shunt study normal	reported; improved 2/2, CR 2/2						
Shah L (2013) [74]	1	22	1/0	Speech	3 d	CT perfusion: hypoperfusion at 3 h, hyperperfusion at 72 h; MRI DWI	
negative	Corticosteroid, valproate; improved 1/1, CR 1/1						
Slavova N (2020) [75]	1	38	1/0	Visual and sensory and speech	4 h	Emergency 1.5/3-T MRI: prominent (index) sulcal draining vein on SWI with ipsilateral hypoperfusion; DWI without infarction	Not reported; improved 1/1, CR 1/1
Thissen S (2014) [76]	2	41, 59	2/0	Visual	3 mo; NR	MRI: no infarction	Acetazolamide, aspirin, lamotrigine, valproate; improved 1/2, CR 1/2
Viola S (2011) [77]	7	21–41	4/3	Speech; Visual and sensory; Visual and sensory and speech; Visual and	8 h; 3 h; 2 h; 7 h; 4 h; 2 h; …	Multimodal: CT/MRI/EEG: hypoperfusion	Aspirin; improved 7/7, CR 7/7

Author (year)	n	Age (years)	F/M	Type of aura	Duration of aura	Investigations	Treatment and outcome
				speech			
Wakakura M (1992) [78]	1	39	1/0	Visual	NR	Goldmann and Humphrey automated static perimetry over time; CT/MRI normal; 123I-IMP SPECT without definite abnormality	Not reported; improved 1/1, CR 0/1
Wang YF (2008) [79]	1	56	1/0	Visual	20 yr	MRI: no infarction	NSAID, flunarizine, lamotrigine, propranolol, topiramate, triptan,
valproate, verapamil; improved 0/0, CR 0/1							
Wolf ME (2018) [80]	1	37	0/1	Visual	2 h	Standardized 3-T protocol with ASL and DSC perfusion during attack and at follow-up: parieto-occipital hyperperfusion, normalized	Not reported; improved 1/1, CR 1/1
Zayat R (2024) [81]	1	57	0/1	Visual and speech	5.5 d	MRI: hyperperfusion, cortical edema	Valproate; improved 1/1, CR 1/1
Zouitina Y (2014) [82]	1	36	0/1	Visual and speech	2 d	MRI: no infarction	Not reported; improved 1/1, CR 1/1
